# Lessons learned and implications of early therapies for coronavirus disease in a territorial service centre in the Calabria region: a retrospective study

**DOI:** 10.1186/s12879-022-07774-9

**Published:** 2022-10-20

**Authors:** Vincenzo Scaglione, Salvatore Rotundo, Nadia Marascio, Carmela De Marco, Rosaria Lionello, Claudia Veneziano, Lavinia Berardelli, Angela Quirino, Vincenzo Olivadese, Francesca Serapide, Bruno Tassone, Helen Linda Morrone, Chiara Davoli, Valentina La Gamba, Andrea Bruni, Bruno Mario Cesana, Giovanni Matera, Alessandro Russo, Francesco Saverio Costanzo, Giuseppe Viglietto, Enrico Maria Trecarichi, Carlo Torti, IDTM U. M. G. COVID-19 Group, Enrico Maria Trecarichi, Alessandro Russo, Francesca Serapide, Bruno Tassone, Paolo Fusco, Vincenzo Scaglione, Chiara Davoli, Rosaria Lionello, Valentina La Gamba, Salvatore Rotundo, Helen Morrone, Lavinia Berardelli, Maria Teresa Tassone, Vincenzo Olivadese, Riccardo Serraino, Chiara Costa, Stefano Alcaro, Caterina De Filippo, Giovambattista De Sarro, Arturo Pujia, Aldo Quattrone, Francesco Saverio Costanzo, Giovanni Cuda, Daniela Patrizia Foti, Giuseppe Viglietto, Giovanni Matera, Federico Longhini, Andrea Bruni, Eugenio Garofalo, Eugenio Biamonte, Vincenzo Brescia, Domenico Laganà, Maria Petullà, Bernardo Bertucci, Angela Quirino, Giorgio Settimo Barreca, Aida Giancotti, Luigia Gallo, Angelo Lamberti, Nadia Marascio, Adele Emanuela De Francesco, Simona Mirarchi, Carlo Torti

**Affiliations:** 1 Chair of Infectious and Tropical Diseases, Department of Medical and Surgical Sciences, “Magna Græcia” University, Viale Europa, Loc. Germaneto, 88100 Catanzaro, Italy; 2 Chair of Clinical Microbiology, Department of Health Sciences, “Magna Græcia” University, Catanzaro, Italy; 3 Department of Experimental and Clinical Medicine, “Magna Græcia” University, Catanzaro, Italy; 4 Chair of Intensive Care, Department of Medical and Surgical Sciences, “Magna Græcia” University, Catanzaro, Italy; 5 Unit of Medical Statistics, Biometrics and Bioinformatics “Giulio A. Maccacaro”, Department of Clinical Sciences and Community Health, Faculty of Medicine and Surgery, University of Milan, Milan, Italy; 6 Department of Experimental and Clinical Medicine, Interdepartmental Center of Services (CIS), Molecular Genomics and Pathology, “Magna Græcia” University, Catanzaro, Italy

**Keywords:** COVID-19, Monoclonal antibodies, Early therapies, Antivirals, Territorial health services

## Abstract

**Background:**

Monoclonal antibodies (mAbs) and antivirals have been approved for early therapy of coronavirus disease (COVID-19), however, in the real-life setting, there are difficulties to prescribe these therapies within few days from symptom onset as recommended, and effectiveness of combined use of these drugs have been hypothesised in most-at-risk patients (such as those immunocompromised) but data supporting this strategy are limited.

**Methods:**

We describe the real-life experience of SARS-CoV-2 antivirals and/or monoclonal antibodies (mAbs) and focus on the hospitalisation rate due to the progression of COVID-19. Clinical results obtained through our risk-stratification algorithm and benefits achieved through a strategic proximity territorial centre are provided. We also report a case series with an in-depth evaluation of SARS-CoV-2 genome in relationship with treatment strategy and clinical evolution of patients.

**Results:**

Two hundred eighty-eight patients were analysed; 94/288 (32.6%) patients were treated with mAb monotherapy, 171/288 (59.4%) patients were treated with antivirals, and 23/288 (8%) patients received both mAbs and one antiviral drug. Haematological malignancies were more frequent in patients treated with combination therapy than in the other groups (p = 0.0003). There was a substantial increase in the number of treated patients since the opening of the centre dedicated to early therapies for COVID-19. The provided disease-management and treatment appeared to be effective since 98.6% patients recovered without hospital admission. Moreover, combination therapy with mAbs and antivirals seemed successful because all patients admitted to the hospital for COVID-19 did not receive such therapies, while none of the most-at-risk patients treated with combination therapy were hospitalized or reported adverse events.

**Conclusions:**

A low rate of COVID-19 progression requiring hospital admission was observed in patients included in this study. The dedicated COVID-19 proximity territorial service appeared to strengthen the regional sanitary system, avoiding the overwhelming of other services. Importantly, our results also support early combination therapy: it is possible that this strategy reduces the emergence of escape mutants of SARS-CoV-2, thereby increasing efficacy of early treatment, especially in immunocompromised individuals.

**Supplementary Information:**

The online version contains supplementary material available at 10.1186/s12879-022-07774-9.

## Background

Since January 2020, global health systems have been fighting the coronavirus disease (COVID-19) pandemic [1–3]. Most people with COVID-19 remain asymptomatic, but in a small proportion of individuals, COVID-19 can rapidly progress and requires hospitalisation and intensive care [4]. Severe disease due to COVID-19 is associated with older age, obesity, and several chronic medical conditions, including cardiovascular, kidney, and pulmonary comorbidities [5, 6]. Emergency authorisation has been provided for several therapies for which a benefit on the course of severe acute respiratory syndrome coronavirus 2 (SARS-CoV-2) infection has been reported [5]. Vaccination has provided an extensive benefit in the prevention of severe COVID-19 [7]; however, its limitations have been extensively described: several patients, especially those affected by immunosuppression, may not be fully protected after vaccination [8] and the emergence of new SARS-CoV-2 variants may impair vaccine efficacy [9, 10].

Neutralizing monoclonal antibodies (mAbs) are a promising treatment to limit disease progression [11–14]. These drugs are human immunoglobulin (Ig)-G1 antibodies that neutralise the virus by binding to the spike protein of SARS-CoV-2 and preventing attachment of the virus to the human cellular receptor angiotensin-converting enzyme 2 [11]. Recently, the early use of three antiviral drugs has been authorised against SARS-CoV-2: remdesivir (RMD), molnupiravir (MOL), and nirmatrelvir/ritonavir (NRM/r) [15–17]. These drugs should be used early, ideally within 5 days from symptom onset [18], determining important consequences on access and linkage to care. Pivotal drug studies of mAbs and antivirals have been focused on non-hospitalised, unvaccinated adults with mild-to-moderate, laboratory-confirmed COVID-19 and at least one risk factor for severe COVID-19 illness [12–17]. Real-life data on patients treated early with mAbs or antivirals are lacking, especially considering the population for whom a combined treatment strategy including both mAbs and antivirals has been suggested [19–21] even if it is not currently recommended [22] because of lack of data. Moreover, the actual benefit of these therapies in low-risk patients is still debated, and overtreatment is a real risk with important consequences related to drug-related adverse events and health costs.

Our hypotheses were that early combination therapy with both mAbs and antivirals improves clinical outcomes in most-at-risk patients identified using an algorithm for risk stratification and that having a dedicated and easily accessible COVID-19 centre is useful to improve both clinical care delivery and to avoid overwhelming the hospital system and emergency territorial service. Therefore, our primary objective was to describe the real-life experience of early therapy for COVID-19 at the University COVID-19 Centre of the Calabria Region (Southern Italy), with a focus on the hospitalisation rate due to the progression of COVID-19. Clinical results obtained through our risk-stratification algorithm at the strategic proximity territorial centre are provided, and a case series on selected and challenging clinical cases is presented.

## Methods

### Study design and population

**Fig. 1:**
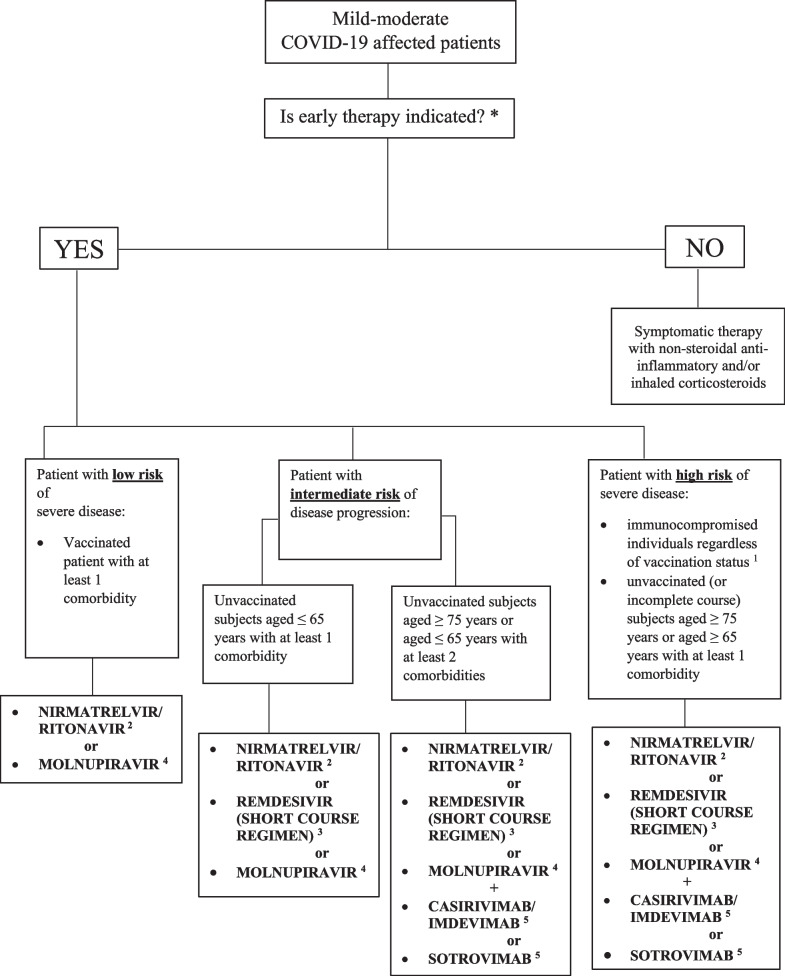
Flow chart of drugs prescription choices according to risk of progression of COVID-19

This retrospective study was conducted at “Mater Domini” University Hospital of Catanzaro, Calabria region (Southern Italy). Patients' risk progression was stratified according to a flow chart (Fig. 1). This algorithm was implemented by our infectious disease team, considering the recently proposed regional guidelines [23, 24].

We included in this study only patients with mild or moderate COVID-19 without the need of incremental support of oxygen receiving early therapy following the indications of the Italian Drug Agency [25, 26]. All patients treated with mAbs from April 27, 2021 to April 2, 2022 were included, as well as all patients treated with antivirals (RMD, MOL, or NRM/r) from January 21, 2022 to April 30, 2022. Moreover, we will also report the clinical outcomes of a selected group of patients who were treated both with mAbs and one antiviral drug.

Despite the lack of data on the combination of mAbs and antiviral therapy in patients affected by COVID-19 [22] and albeit it was not recommended by two operational procedures, including that adopted in the Calabria region [23, 24], this strategy was applied in our algorithm based on the rationale of reducing the escaping mutants of the SARS-CoV-2, and considering preliminary observations and cases, which were subsequently published [19–21, 27, 28], indicating possible increased efficacy.

### Setting in the territorial service centre

Calabria region includes five provinces of which Catanzaro, located in the central area, is the third for number of inhabitants (about 350,000). In the Calabria region, 10 and 8 territorial prescribing centres for antiviral and mAb therapies, respectively, were established for high-risk patients with mild to moderate COVID-19 [29].

**Fig. 2:**
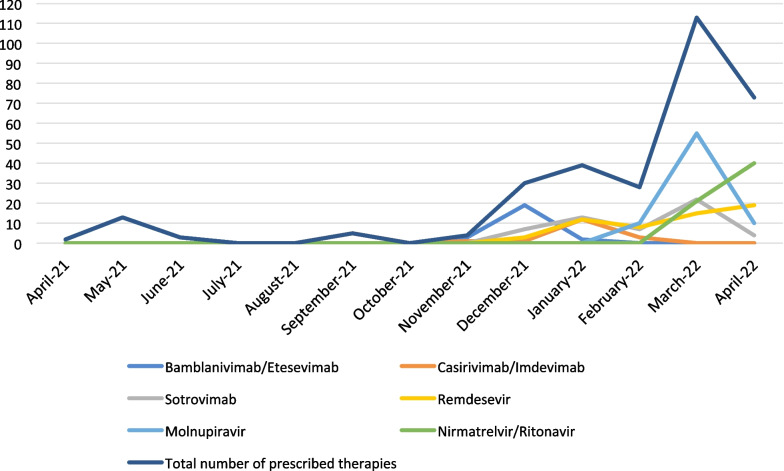
Number of patients affected by mild-moderate COVID-19 treated over time and relative number of prescribed drugs

According to the regional organisation system, our centre was dedicated to patients residing in the Catanzaro province, who tested positive for SARS-CoV-2 and referred by a local medical territorial service (i.e., a COVID-19-dedicated territorial physician or patients’ general medicine doctor). Moreover, our centre was also available for patients coming from other provinces for clinical evaluation and therapy as a pilot, regional, referral centre. Since March 2, 2022, when the centre was opened, an increase in the number of treated patients was observed compared to the previous period (Fig. 2). As at the end of April 2022, about 7,500 patients were affected by SARS-CoV-2 infection in the Catanzaro province, most of them (98.8%) not hospitalised. In the same period, in the rest of the region, among 1,500,000 inhabitants, there were about 78,000 prevalent cases of SARS-CoV-2 infection.

Fifteen percent of the mAbs and 12.9% of the antivirals delivered to the Calabria region were prescribed at our centre. mAbs and antivirals were prescribed according to the Italian Drug Agency criteria [25, 26], considering the local variant epidemiology over time and drug availability. Figure 1 depicts a flow chart of drug prescription choices [23, 24]. For outpatients, a dedicated telephone number was available for use in cases of clinical worsening before proceeding to hospital admission. Proactive telephone monitoring was performed weekly by our infectious disease team until the achievement of SARS-CoV-2 negative molecular/third-generation rapid antigen nasopharyngeal swab test results.

### Data collection

The following data were collected from patients’ clinical records at baseline (i.e., at the time of clinical evaluation for eligibility): age, sex, body mass index (BMI), underlying disease, date of illness onset, SARS-CoV-2 vaccination status, SARS-CoV-2 infection characteristics, baseline clinical severity according to the National Institutes of Health severity scale score [30], number of days between symptom onset and diagnosis, and number of days between symptom onset and mAb treatment. Types of mAbs and antivirals, as well as adverse reactions were collected.

### Microbiological procedures

As recommended by Italian health authorities [31], a diagnosis of SARS-CoV-2 infection was determined either with a rapid third-generation SARS-CoV-2 antigen nasopharyngeal swab test or real-time polymerase chain reaction (PCR) SARS-CoV-2 molecular test. Molecular test was performed by Allplex™ SARS-CoV-2 Assay (Seegene), detecting Ct values for the Envelope (E), RNA-dependent RNA polymerase/Spike (RdRp/S) and nucleocapsid (N) coding genes.

### SARS-CoV-2 genome analysis by whole genome sequencing

SARS-CoV-2 genomic substitutions were identified in a challenging case series (described later). Longitudinal nasopharyngeal swabs were collected at baseline and during the COVID-19 pandemic. SARS-CoV-2 RNA was extracted using NUCLISENS® easyMAG® (bioMérieux, Florence, Italy), and whole-genome sequencing (WGS) was performed using the Ion S5™ System (Thermo Fisher Scientific, San Diego, CA). Seven microliters of viral RNA were retrotranscribed using the Invitrogen™ SuperScript™ VILO cDNA Synthesis Kit (Thermo Fisher Scientific). Libraries were prepared using the Ion AmpliSeq SARS-CoV-2 Insight Research Panel (Thermo Fisher Scientific) which consisted of 2 primer pools targeting 237 amplicons ranging from 125 to 275 bp in length and tiled across the SARS-CoV-2 genome, with an additional 5 primer pairs targeting human expression controls. Library preparation was performed manually using the Ion AmpliSeq Library Kit Plus (Thermo Fisher Scientific). The final concentration of each complementary (c)-DNA library was determined using the Agilent 2100 System by the Agilent High Sensitivity DNA Assay (Agilent Technologies, Santa Clara, CA), following the manufacturer’s recommendations. Barcoded libraries were diluted to 30 pM and then loaded onto the Ion Chef™ Instrument (Thermo Fisher Scientific) for emulsion PCR, enrichment, and loading onto the Ion S5 520 chip. Post-sequencing run analysis was performed in Ion Torrent Suite Software with the following plugins: SARS-CoV-2_coverageAnalysis to visualize coverage over targeted regions of the SARS-CoV-2 reference genome (Additional file 1: Table S1) and generate Consensus to obtain consensus sequence for each barcode by assembling viral sequences relative to the reference genome of Wuhan-Hu-1 (NC_045512.2) and COVID19AnnotateSnpEff for variant annotation. A complete list of nucleotide changes identified in this study is provided in Additional file 2: Table S2. Variant classification based on whole-genome sequences was performed using the Pangolin tool, version 4.0.6 [32].

### Statistical analysis

Patients were stratified into three groups according to the prescribed therapy (mAbs, antivirals, and combined mAbs and antiviral therapy). Differences between the three groups were assessed. Categorical variables (i.e., comorbidities) were evaluated using the chi-square test or Fisher’s exact test (two-tailed). Values are expressed as the mean ± standard deviation (continuous variables) or as frequencies (absolute and percentage). A p-value < 0.05 was considered statistically significant. All analyses were performed using the statistical software package SAS® (SAS Institute Inc., Cary NC, USA) version 9.4.

## Results

### Demographic, clinical characteristic, treatment and outcome

In this study, 288 patients were analysed; 151 (52.4%) were men and 137 (47.6%) were women, with a mean age of 63.6 (± 16.1) years. Most patients were from the Catanzaro province (234/288, 81.2%). Since the opening of the centre dedicated to early therapies for COVID-19, there was an increase in the number of patients (Fig. 2), including those coming from other provinces of the Calabria region (16/288 from Vibo Valentia; 15/288 from Cosenza; 14/288 from Crotone; and 9/288 from Reggio Calabria). Table 1 displays the socio-demographic characteristics, comorbidities, clinical characteristics, prescribed therapies, and outcome of SARS-CoV-2-infected patients.

**Table 1:** Socio-demographic, co-morbidities, clinical characteristics, prescribed therapies and outcome of SARS-CoV-2-infected patients

Characteristic	All patients n = 288 (%)	Patients treated only with mAbs n = 94	Patients treated only with antivirals n = 171	Patients treated with both mAbs and antivirals n = 23
Gender n (%)				
Male	151 (52.4%)	50 (53.2%)	87 (50.9%)	14 (60.9%)
Female	137 (47.6%)	44 (46.8%)	84 (49.1%)	9 (39.1%)
Age				
Mean (SD)	63.6 (± 16.1)	67 (± 15.1)	62.4 (± 16.5)	58.3 (± 15.8)
65 years or older n (%)	158 (54.9%)	59 (62.8%)	91 (53.2%)	8 (34.8%)
Mean BMI (SD)	28.3 (± 6.1)	29.2 (± 6.6)	27.8 (± 5.7)	27.8 (± 5.8)
Comorbidities n (%)				
Cardiac insufficiency	14 (4.9%)	4 (4.3%)	8 (4.7%)	2 (8.7%)
Cerebral ischemia history	28 (10.9%)	13 (13.8%)	13 (7.6%)	2 (8.7%)
COPD	53 (18.4%)	13 (13.8%)	37 (21.6%)	3 (13%)
Autoimmune diseases	36 (12.5%)	7 (7.4%)	25 (14.6%)	4 (17.4%)
Vasculopathy	24 (8.3%)	12 (12.8%)	9 (5.3%)	3 (13%)
Hepatic cirrhosis	6 (2.1%)	1 (1.1%)	5 (2.9%)	0
Obesity	91 (31.6%)	35 (37.2%)	51 (29.8%)	5 (21.7%)
Diabetes				
Compensated disease	63 (21.9%)	24 (25.5%)	36 (21.1%)	3 (13%)
Decompensated disease	10 (3.5%)	3 (3.2%)	6 (3.5%)	1 (4.3%)
Chronic kidney diseases	19 (6.6%)	9 (9.6%)	7 (4.1%)	3 (13%)
Solid cancer				
Localized disease	34 (11.8%)	6 (6.4%)	23 (13.5%)	5 (21.7%)
Metastatic disease	15 (5.2%)	3 (3.2%)	12 (7.1%)	0
Hematological malignancies	43 (14.9%)	10 (10.6%)	23 (13.5%)	10 (43.5%)
AIDS	1 (0.3%)	1 (1.1%)	0	0
History of peptic ulcer	2 (0.7%)	0	2 (1.2%)	0
Ischemic heart diseases	36 (12.5%)	15 (16%)	17 (9.9%)	4 (17.4%)
Dementia	17 (5.9%)	10 (10.6%)	7 (4.1%)	0
Hemiplegia	3 (1%)	1 (1.1%)	1 (0.6%)	1 (4.3%)
Hypertension	165 (57.3%)	60 (63.8%)	93 (54.4%)	12 (52.2%)
Number of comorbidities ≥ 3	113 (39.2%)	44 (46.8%)	60 (35.1%)	9 (39.1%)
Charlson Comorbidity Index (mean ± SD)	4.1 (± 2.3)	4.2 (± 2.2)	4 (± 2.3)	4.1 (± 2.2)
Vaccination				
Yes	232 (82.3%)	56 (59.6%)	161 (94.2%)	18 (78.3%)
No	51 (17.7%)	38 (40.4%)	10 (5.8%)	5 (21.7%)
Mean days from symptoms onset to first positive swab (SD)	1.3 (1.4)	1.7 (1.7)	1.2 (1.2)	1.1 (1.1)
Mean days from symptoms onset to treatment (SD)	3.3 (1.9)	3.9 (2.4)	3.1 (1.5)	3.5 (2.2)
Baseline clinical severity n (%)				
ASY	47 (16.3%)	39 (41.5%)	3 (1.8%)	5 (21.7%)
MID	235 (81.6%)	52 (55.3%)	167 (97.6%)	16 (69.6%)
MOD	6 (2.1%)	3 (3.2%)	1 (0.6%)	2 (8.7%)
IgG anti-SARS-CoV-2-Spike protein at baseline n (%)				
Positive	70 (24.3%)	45 (47.9%)	18 (10.5%)	7 (30.4%)
Negative	65 (22.6%)	46 (48.9%)	4 (2.3%)	15 (65.2%)
N/A	153 (53.1%)	3 (3.2%)	149 (87.1%)	1 (4.3%)
Monoclonal antibodies n (%)*				
Bamlanivimab/Etesevimab	42 (35.9%)	40 (42.6%)	–	2 (8.7%)
Casirivimab/Imdevimab	22 (18.8%)	17 (18.1%)	–	5 (21.7%)
Sotrovimab	53 (45.3%)	37 (39.4%)	–	16 (69.6%)
**Total number	117	94	–	23
Antivirals n (%)*				
Remdesivir	57 (29.1%)	–	38 (22.2%)	18 (78.3%)
Molnupiravir	77 (39.8%)	–	75 (43.9%)	2 (8.7%)
Nirmatrelvir/Ritonavir	61 (31.1%)	–	58 (33.9%)	3 (13%)
**Total number	195	–	171	23
COVID-19 progression and related hospital admission				
Yes	4 (1.4%)	3 (3.2%)	1 (0.6%)	0
No	284 (98.6%)	91 (96.8%)	170 (99.4%)	23 (100%)

*SD* standard deviation; *COPD* chronic obstructive pulmonary disease; *ASY* asymptomatic or pre-symptomatic; *MID* mild; *MOD* moderate; *COVID-19* Coronavirus Disease 2019

*Percentage is calculated on total number of vaccinated patients as indicated appropriately in each column

**Percentage is calculated on total number of prescribed mAbs and antivirals as indicated appropriately in each column

Diagnosis was made after a mean of 1.3 (standard deviation, SD: 1.4) days from symptom onset. At the clinical evaluation, most patients had mild disease (235/288, 81.6%). The most prevalent comorbidities were hypertension (165/288, 57.3%), obesity (91/288, 31.6%), compensated diabetes mellitus (63/288, 21.9%), and chronic obstructive pulmonary disease (53/288, 18.4%). Patients were stratified into three groups: patients treated with mAb monotherapy, 94/288 (32.6%); patients treated with antiviral therapies, 171/288 (59.4%); and patients treated with both mAbs and one antiviral therapy, 23/288 (8%). No comorbidities were statistically different between the three groups except for haematological malignancies, which were more frequent in patients treated with combined mAbs and antiviral therapy than in the mAb monotherapy and antiviral therapy groups (18/23, 78.3% versus [vs.] 23/171, 13.5% vs. 10/94, 10.6%; p = 0.0003), and neurocognitive impairment disorders, which were more frequent in patients treated with mAb monotherapy than in the other groups (10/94, 10.6% vs. 7/171, 4.1% vs. 0/23; p = 0.044). Overall, 113/288 (39.2%) patients had at least 3 comorbidities. Most patients received the main course of the SARS-CoV-2 vaccine (81.9%), and 55.2% also received the booster dose. The mean number of days from the last dose to SARS-CoV-2 infection was 118.5 (SD: 66.3).

Treatment was administered after a mean of 3.3 (SD: 1.9) days from symptom onset. None of the patients died, except for two of them, both receiving mAbs (the first due to complications of atrial fibrillation and the second for late complications of COVID-19 pneumonia). Sotrovimab (SOT) was the most frequently prescribed mAb, whereas MOL was the most frequently prescribed antiviral. Clinical worsening was observed in 4 patients who required hospital admission: 1 patient treated with bamlanivimab/etesevimab (BAM/ETE), 2 patients treated with SOT and 1 patient treated with RMD (see *cases #1–4* described below).

Regarding patients treated with both antiviral and mAbs, most (18/23, 78.3%) were affected by immunocompromising conditions: 10/23 (43.5%) were affected by haematological malignancies, 4/23 (17.4%) were affected by autoimmune diseases, 2/23 (8.7%) were affected by active solid tumors, and 2/23 (8.7%) were renal transplanted with ongoing immunosuppressive therapy. No patients had previously received prophylaxis with tixagevimab/cilgavimab, since this combination of mAbs was not yet authorized in Italy for this indication. Seven of these 23 patients (30.4%) had been previously treated with anti-CD20 mAbs (6 patients with rituximab and 1 patient with ocrelizumab), and 2 patients (8.7%) received adalimumab and daratumumab, respectively. Most patients had already received the primary SARS-CoV-2 vaccination cycle (18/23, 78.3%), and 7/18 (38.9%) had already received the third dose (i.e., the booster dose); in 15/23 (65.2%) patients in this subgroup, the IgG anti-SARS-CoV-2-spike protein was negative. None of these patients treated with mAbs in combination with antiviral therapy required hospital admission. In particular, one patient treated with combination treatment obtained a negative molecular test for SARS-CoV-2 RNA on nasopharyngeal swab with resolution of symptoms very rapidly (see *case #5* described below).

Overall, one patient experienced an urticarial skin rash after treatment with MOL; therefore, treatment was discontinued. Four patients stopped NRM/r during treatment because of vomiting and/or diarrhoea that did not respond to medical therapy. One patient reported general illness, paleness, and asthenia following the SOT infusion, and an allergic reaction to the drug was suspected. Corticosteroid and antihistamine therapy was administered with clinical improvement. None of the patients treated with combined therapy had side effects.

### Clinical and virological course of patients who received monotherapy as initial treatment for COVID-19

Table 2 summarises the main features of cases treated with mAbs and/or antivirals. Overall, these cases suggested genomic evolution in a patient with long lasting infection who needed several courses of therapy, compared with the last patient who achieved a very rapid resolution of the infection after initial treatment with combined therapy. For the remaining patients not treated initially with combination therapy, several courses of treatment appeared to be necessary.

**Table 2:** Clinical features of patients treated with mAbs and/or antivirals

Patient number	Gender, age	Comorbidities	COVID-19 vaccination	Baseline IgG anti-spike protein of SARS-CoV-2	Current or previous (within 6 months) use of immune-suppressive drugs	Initial COVID-19 therapy (time from symptom onset)	Length of hospitalisation (days)	Subsequent COVID-19 therapies, (time from symptom onset)	Clinical complications	Clinical course and outcome
1	F, 60 years	Autoimmune anaemia due to systemic lupus erythematosus	2 doses of mRNA vaccine	Negative	Rituximab	BAM/ETE (5 days)	37 days	CAS/IMD and RMD (47 days)	Respiratory failure Severe anaemia Non-ST-elevation myocardial infarction Secondary depressive syndrome	Improvement of haemoglobin levels was observed after SARS-CoV-2 RNA negativisation on naso-pharyngeal swab which occurred after 12 days from CAS/IMD and RMD. The patient was subsequently discharged
2	M, 73 years	Low-grade non-infiltrating papillary urothelial bladder cancer Type 2 diabetes mellitus hypertension hypercholesterolemia Kidney transplant	3 doses of mRNA vaccine	Negative	Mycophenolate mofetil tacrolimus	SOT (5 days)	40 days	None	Severe respiratory failure diarrhoea	Complications resolved and the patient was discharged continuing low-flow oxygen therapy
3	M, 38 years	Tetraparesis due to multiple sclerosis	No	Negative	-	SOT (2 days)	8 days	RMD (6 days)	Severe respiratory failure	Complication resolved and the patient was discharged after 8 days since RMD
4	M, 56 years	Severe obesity Type 2 diabetes mellitus Hypertension Non-Hodgkin lymphoma	2 doses of mRNA vaccine	Negative	Rituximab	RMD (5 days)	54 days	CAS/IMD (16 days)	Severe respiratory failure	Complication resolved and the patient was discharged after 54 days since CAS/IMD continuing low-flow oxygen therapy
5	F, 41 years	Burkitt lymphoma	2 doses of mRNA vaccine	Positive	R-HYPER-C-VAD	SOT and RMD (4 days)	3 days	None	None	The patient was discharged home soon after SOT and RMD and negativisation of SARS-CoV-2 RNA on naso-pharyngeal swab occurred after 6 days from SOT and RMD

*F* female; *M* male; *BAM/ETE* bamlanivimab/etesevimab; *SOT* sotrovimab; *RMD* remdesivir; *CAS/IMD* casirivimab/imdevimab; *R-HYPER-C-VAD* rituximab, cyclophosphamide, vincristine, adriamycin and dexamethasone; mRNA, Messenger RNA.

Patients who received oxygen support by non-invasive ventilation or high-flow nasal cannula were considered to be affected by severe respiratory failure

#### Case #1

A 60-year-old woman treated with rituximab for severe autoimmune anaemia due to systemic lupus erythematosus on May 27, 2021 developed fever, cough, sore throat, ageusia, and anorexia, and was diagnosed with SARS-CoV-2 infection. Despite vaccination against SARS-CoV-2, the IgG anti-SARS-CoV-2-spike protein at baseline was negative. On June 3, 2021, mAb therapy with BAM/ETE (700/1400 mg) was administered. On June 10, 2021, she was admitted to our COVID-19 Centre for fever and dyspnoea, and empirical antibiotic therapy with piperacillin/tazobactam was started. On June 22, 2021, productive cough and X-ray signs of pneumonia led to antibiotic therapy with meropenem. During her hospital stay, severe and progressive anaemia not responsive to iron sulfate and folic acid occurred, and a positive Coombs test result contraindicated a blood transfusion. Hence, therapy with erythropoietin, corticosteroids, and danazol was started, and the haemoglobin levels showed a partial response. Moreover, severe anaemia and coronary stenosis led to non-ST-elevation myocardial infarction (NSTEMI) which was treated with aspirin, clopidogrel, and enoxaparin. Low-flow oxygen therapy was administered because of respiratory failure caused by NSTEMI. Molecular nasopharyngeal swabs for SARS-CoV-2 were performed weekly resulting persistently positive. Both casirivimab/imdevimab (CAS/IMD) and RMD were administered 35 days after the first mAb course. The first negative molecular nasopharyngeal swab result was detected 77 days after the first administration of mAbs, and consequently, improvement in haemoglobin levels was observed.

**Fig. 3:**
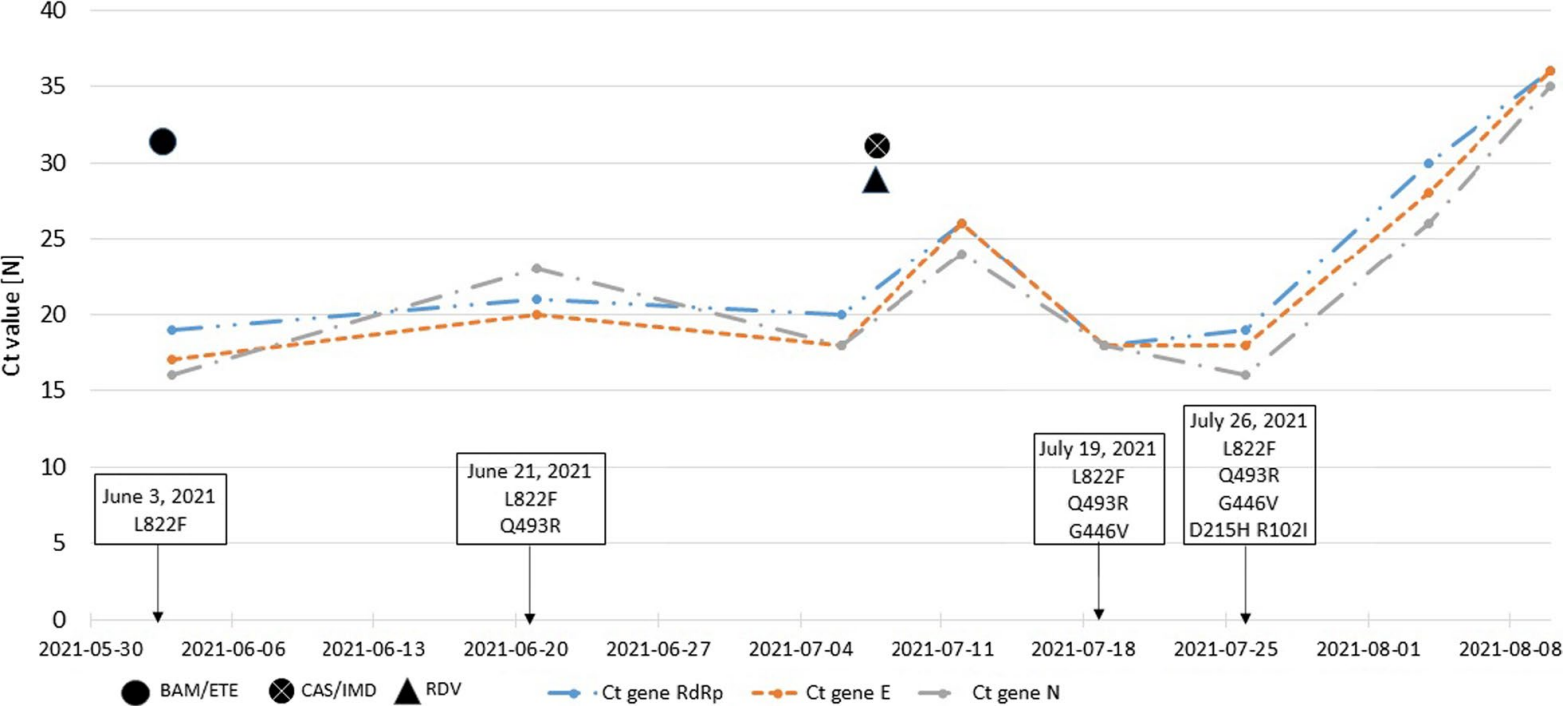
Cycle threshold (Ct) of RNA-dependent RNA polymerase (RdRp), envelope (E) and nucleocapsid (N) genes and mutations occurred over time in case #1

In this case of slow-resolution SARS-CoV-2 infection and multiple clinical complications, we collected nasopharyngeal swabs from the first mAb administration until the first negative nasopharyngeal swab result at 8 different time points (Fig. 3). The viral genomes isolated from nasopharyngeal swabs were analysed using WGS. All viral isolates shared a common core mutation typical of the B.1.1.7 variant of concern (VOC). Several mutations occurred in the major (> 90%) and minor (< 90%) viral populations during follow-up, as shown in Table 3 and Fig. 3. At baseline, the viral isolate was characterized by the deletion of 144-146del in the S gene. After mAb therapy, mutations that were not naturally present in the B.1.1.7 VOC were observed. On June 21, 2021, 18 days after BAM/ETE administration, the Q493R mutation in the RBD of Spike protein was selected and maintained at more than one follow-up time point. On July 12, 2021, the major viral population carried the Q493R mutation, and the minor viral population had the G446V (42%) mutation. On July 19, 2021, the G446V mutation characterised the major population (93%) of the SARS-CoV-2 isolate, whereas the R246I mutation was observed in the minor viral population (27%). In the follow-up samples, the R246I mutation was no longer detected, whereas the G446V and Q493R mutations were maintained. Notably, on August 4, 2021, mutations other than A67S appeared in a minor viral population (21%). Notably, from July 19, 2021, the E484Q mutation was detected in the minor viral population at a frequency of 62%, becoming prevalent until the last amplifiable sample.

**Table 3:** Analysis of mutation profile of newly characterized SARS-CoV-2 isolates at baseline and during therapy of selected patients

Patient number	Collection date	VOC	S gene		ORF1ab	E	M	ORF7a		N
			AF > 90%	AF < 90%	AF > 90%	AF < 90%	AF > 90%	AF > 90%	AF < 90%	AF > 90%
1	2021–06-03	B.1.1.7	L822F		P1950L,A5376V, K5784R		V70L	P34L		P279Q
	2021–06-21	B.1.1.7	Q493R, L822F		P1950L,A5376V, K5784R		V70L	P34L		P279Q
	2021–07-06	B.1.1.7	Q493R, L822F	A243V (61%), L938F (47%)	P1950L,A5376V, K5784R		V70L	P34L		P279Q
	2021–07-12	B.1.1.7	Q493R, L822F	A243V (25%), G446V (42%), L938F (38%)	P1950L,A5376V, K5784R		V70L	P34L		P279Q
	2021–07-19	B.1.1.7	G446V, Q493R, L822F	R102I (64%) R246I (27%), E484Q (62%)	P1950L,A5376V, K5784R	V62F (28%)	V70L	P34L		P279Q
	2021–07-26	B.1.1.7	R102I, D215H, G446V, E484Q, Q493R, L822F		P1950L,A5376V, K5784R		V70L	P34L		P279Q
	2021–08-04	B.1.1.7	R102I, D215H, G446V, E484Q, Q493R, L822F	A67S (21%), G252V (69%), T573I (26%)	P1950L,A5376V, K5784R		V70L	P34L		P279Q
2	2022–01-21	BA.1.1.529 (BA.1.17)			P1803S					
	2022–02-12	BA.1.1.529 (BA.1.17)			P1803S					
3	2022–03-28	BA.1.1.529 (BA.2.9)			A2279V, L6404I					
4	2022–05-26	BA.1.1.529 (BA.2)							P34L (40%)	P326L (77%)
5	2022–01-09	BA.1.1.529 (BA.1.15)								P383L

*VOC* Variant of concern; *AF* Allele frequency

#### Case #2

A 73-year-old man with low-grade non-infiltrating papillary urothelial bladder cancer, type 2 diabetes mellitus, hypertension, and hypercholesterolemia who underwent kidney transplant due to severe renal failure 17 years ago received SOT (500 mg/ev) on January 21, 2022 because of pauci-symptomatic COVID-19. Although the patient had already received vaccine against SARS-CoV-2, he had undetectable IgG anti-SARS-CoV-2-spike antibodies at baseline. From January 30, 2022, clinical worsening with high grade fever and diarrhoea occurred; thus, the patient required admission on February 2, 2022. During hospitalisation, progressive respiratory failure occurred, so the patient required high-flow nasal cannula oxygen therapy and non-invasive ventilation (NIV).

From February 4, 2022, drowsiness and slight tremor affecting the upper limbs were observed; therefore, brain CT and lumbar puncture were performed. Microbiological and chemical examinations performed on the cerebrospinal fluid were all negative as were examinations performed on stool. From February 8, 2022, due to persistent fever and further clinical worsening, antibiotic therapy with meropenem, linezolid and caspofungin was started; mycophenolate was replaced with prednisone (10 mg/day) and isavuconazole was added considering possible invasive pulmonary aspergillosis.

From February 17, 2022, progressive clinical and respiratory improvements were observed and antibiotic therapy was no longer necessary. On February 21, 2022, the molecular nasopharyngeal swab resulted negative and on March 2, 2022, he was discharged with an indication to continue oxygen therapy (2 L/min). The patient was infected with the B.1.1.529 VOC sub-lineage BA.1.17. The WGS analysis was performed at baseline and 32 days after SOT administration. In both isolates, sub-lineage BA.1.17 displayed the typical spike mutations of the B.1.1.529 VOC (Table 3).

#### Case #3

On March 23, 2022, a 38-year-old man with tetraparesis due to multiple sclerosis not previously vaccinated against SARS-CoV-2 developed a fever; subsequently, he and his caregiver were tested for SARS-CoV-2 through a nasopharyngeal swab, which showed a positive result. SOT (500 mg/ev) was administered on March 28, 2022. On March 29, 2022, the patient’s caregiver reported worsening of peripheral oxyhaemoglobin saturation, which reached 84% on room air; therefore, the patient was admitted to the hospital where low-flow oxygen therapy, anticoagulant prophylaxis with enoxaparin and methylprednisolone, and antiviral therapy with RMD were administered. On March 30, 2022, further clinical worsening requiring NIV was observed. Blood cultures were performed and empirical antibiotic therapy with piperacillin/tazobactam plus levofloxacin was added. On March 31, 2022, clinical improvement and resolution of the respiratory failure were observed. Blood culture resulted negative and thorax CT scan showed interstitial pneumonia. Antiviral, anticoagulant, corticosteroid, and antibiotic therapies were continued until discharge (April 5, 2022).

The patient’s viral isolate presented a typical core mutation common of B.1.1.529 VOC sub-lineage BA.2.9. Table 3 shows the mutations discriminated by the patient’s swab, and Additional file 1: Table S1 presents the complete list of mutations characterising the viral isolate.

#### Case #4

A 56-year-old man with severe obesity (BMI, 47 kg/m^2^), type 2 diabetes mellitus treated with metformin, hypertension, and non-Hodgkin lymphoma treated with rituximab was admitted on April 11, 2022 for early therapy against COVID-19. A short course (3 days) of RMD and inhalation therapy with budesonide (400 mg twice a day) was prescribed. Despite vaccination against SARS-CoV-2, serological test results at baseline were negative. Progressive clinical worsening characterised by cough, fever, and respiratory failure was observed; hence, on April 21, 2022, he was admitted to the hospital. On April 22, 2022, mAb therapy with CAS/IMD (1200/1200 mg) was administered and empirical antibiotic therapy with piperacillin/tazobactam and levofloxacin was initiated. On May 5, 2022, bronchoscopy was performed because of persistent respiratory failure and antibiotic therapy was modified with meropenem and linezolid. Respiratory function slowly improved and on June 14, 2022 the patient was discharged, continuing oxygen therapy (2 L/min) at home. The patient’s viral isolate showed a common core mutation typical of the B.1.1.529 VOC sub-lineage BA.2, including S371F, D405N, K417N, E484A, and Q493R mutations. Table 3 presents the mutations discriminated by the patient’s swab, and Additional file 2: Table S2 shows the complete list of viral mutations.

### Clinical and virological course of a patient treated with early combination therapy for COVID-19

#### Case #5

As summarized in Table 2, a 41-year-old woman with Burkitt lymphoma treated with 3 cycles of R-HYPER-C-VAD (rituximab, cyclophosphamide, vincristine, adriamycin, and dexamethasone) developed sore throat, arthromyalgia, and malaise, and on January 5, 2022, she was diagnosed with SARS-CoV-2 infection. On January 9, 2022, she was admitted for clinical evaluation. Despite having received only 2 doses of a mRNA vaccine (the last 1 year before), the IgG anti-SARS-CoV-2-spike protein was positive. According to a previously reported flow chart, we proposed combined therapy with SOT and RMD considering her clinical conditions and the incomplete vaccination cycle against SARS-CoV-2. Clinical improvement was rapidly observed, and on January 12, 2022, she was discharged home. After three days from discharge, a molecular nasopharyngeal SARS-CoV-2 swab resulted negative.

The patient was infected with sub-lineage BA.1.15, which displayed the common core spike mutations of the B.1.1.529 VOC. Table 3 displays the characteristic mutations observed for the SARS-CoV-2 viral isolate of this patient, and Additional file 2: Table S2 shows the complete list of mutations of the viral isolate.

## Discussion

The aim of the present study was to describe real-life experiences on the use of early therapies for COVID-19 in high-risk SARS-CoV-2-infected patients at the University COVID-19 Centre of Calabria region. To our knowledge, this is the largest Italian cohort of patients with COVID-19 who were treated early with mAbs and/or antivirals. Our experience demonstrated how proximity to a COVID-19-dedicated centre may improve the access to care facilitating successful clinical management. Moreover, by applying our algorithm for risk stratification, disease management and treatment, hospital admission was not required for most patients (98.6%).

Several factors may have contributed to this success. (i) Vaccine efficacy: most patients had already received a full schedule course against SARS-CoV-2 and were protected against severe COVID-19 [33, 34]. However, vaccinated people may present risk factors for disease progression [35], such as advanced age and comorbidities, which were more represented in our cohort compared with patients included in pivotal clinical trials [12–17]. Indeed, most patients were elderly (54.9%) and had at least 3 comorbidities (39.2%). Additionally, the length between the last dose of the vaccine and COVID-19 onset is a critical point in assessing the protection of patients against the severe form of the disease; the longer the length, the higher the risk of having a lower level of immunity and protection [36]. In the present study, patients who received mAb monotherapy also received the COVID-19 vaccine several months before SARS-CoV-2 infection (mean time: 153 days). Lastly, a significant benefit of early use of antivirals (namely NRM/r) was demonstrated even in vaccinated patients with a high risk of clinical complications, leading to a 38% lower risk of severe COVID-19 or death compared to placebo, when omicron was the dominant SARS-CoV-2 variant [37]. (ii) Rapid clinical assessment: diagnosis of SARS-CoV-2 infection was performed after a mean time of 1.3 days from symptom onset, and therapies were administered after a mean of 3.3 days. We also highlighted the capacity of our territorial service centre, since 186/288 (64.6%) patients were managed as outpatients in only 2 months of activity, while 102/288 (35.4%) were admitted to inpatient care during the 10 months of the study preceding the opening of the territorial centre to administer the treatment, often without any other appropriate indication for the hospital admission; therefore, implementation of territorial service centres could avoid hospital overcrowding, leading to possible clinical, epidemiological and pharmacoeconomical benefits. (iii) Antiviral therapies: most patients received antivirals (67.7%), which showed in vitro activity against all SARS-CoV-2 VOCs [38]. We believe that antiviral broad-spectrum activity against all SARS-CoV-2 variants may have had a further protective effect on the analysed patients, potentially affected by further vaccine-escaped SARS-CoV-2 variants [39] or they did not respond to the vaccine [10]. However, owing to the lack of a control group, we could not fully explore this hypothesis. (iv) Antiviral and mAb combination therapy: most patients included in this subgroup were immunosuppressed because of haematological malignancies (43.5%) or other clinical conditions. Despite the fact that most patients received full vaccination for SARS-CoV-2, 65.2% of IgG anti-SARS-CoV-2-spike antibodies were negative. We believe that combined therapy with mAbs and antiviral therapy may have had a benefit on clinical outcomes, as reported by other authors [19–21], as no patient treated with both mAbs and antiviral therapy required hospital admission.

We also emphasize that all patients for which COVID-19 progression was observed were immunocompromised hosts, and after a long “honeymoon” period characterized by apparent clinical stability, clinical worsening occurred. Clinical management of these patients should be better tailored, and prompt treatment should be performed [20]. Conversely, in case #5, despite severe immunosuppression, after combination therapy with SOT and RMD, the patient rapidly improved and obtained viral clearance from naso-pharyngeal swab.

Cases #1 to #4 illustrated clinical worsening after treatment with mAbs (cases #1 to #3) or antiviral (RMD) (case #4). All viral isolates from these cases were sequenced, and several mutations were detected. In case #1, deletion of 144-146del in the S gene was observed at baseline. This deletion has been associated with resistance to several NTD-binding neutralising mAbs [40]. The Q493Ris mutation has been associated with long SARS-CoV-2 infections [41] and increased (≥ 25 fold) resistance to BAM/ETE and CAS [40, 42]. The G446V mutation subsequently observed in a major population (93%) of SARS-CoV-2 isolates may contribute to the increased viral resistance to IMD (≥ 25 fold), while its effect on susceptibility to CAS may not be significant [43].

The same is true for the R246I mutation observed in the minor viral population (27%), which is generally observed for the B.1.351 VOC [40], also it has been shown to be associated with resistance to several neutralizing mAbs binding to the N-terminal domain (NTD) [40]. Notably, on August 4, 2021, the A67S mutation was detected in the minor viral population (21%), which was recently observed in the B.1.1.7 VOC in the Calabria region and was associated with impaired recognition by T cells [44]. Lastly, the E484Q mutation reduces susceptibility to BAM (> 100 fold), and CAS (approximately tenfold) has already been reported [45]. Importantly, under mAbs pressure Spike protein selected the Q493R mutation, which later became natural mutation of Omicron VOC, suggesting a viral fitness advantage.

Regarding mutations of the ORF1a/b gene region, to our knowledge, no association with mAb therapy has been reported. By contrast, Gandhi et al. reported that treatment with RDV may favor the E802D mutation in the nsp12 protein encoded by ORF1b [46]; however, in case #1, RDV combined with CAS/IMD did not result in the selection of nsp12 E802D.

Case #2 was infected with B.1.1.529 VOC sub-lineage BA.1.17. The G339D and S371L mutations have been associated with an elevated escape fraction and reduced susceptibility to SOT [47], whereas the 143-145del mutation was associated with resistance to several neutralizing mAbs [40]. In contrast, the N440K mutation, typical of the B.1.1.529 VOC, was not associated with resistance to SOT [48].

In case #3 at baseline, SARS-CoV-2 (BA.2.9 sub-lineage) major viral population usual spike mutations, such as G339D, S371L, and N440K were observed, for which, as reported earlier, impairment or maintenance of susceptibility to SOT, respectively, has been described [47, 48].

The S371F mutation observed in the SARS-CoV-2 (BA.2.9 sub-lineage) isolate from case #4 was associated with impairment of CAS (14 to 28 fold) and IMD (11 to 126 fold) [47], whereas D405N and K417N mutations reduced susceptibility to CAS [49]. E484A and Q493R mutations were associated with resistance selection in vivo by CAS/IMD mAbs and high-level resistance to CAS, respectively [42, 50].

Case #5 was infected with sub-lineage BA.1.15, which displayed the common core spike mutations of the B.1.1.529 VOC. As reported for other B.1.1.529 VOC-positive patients, the SARS-CoV-2 major viral population carried G339D, S371L, 143-145del, and N440K mutations related to SOT-administered therapy [40, 47, 48]. Based on these observations, it is reasonable that combination therapy (including both antivirals and mAbs) should be performed as soon as possible in immunocompromised patients to control viral replication, hampering the emergence of escaping mutans and improving the clinical outcome.

This study has several limitations. First, it is a retrospective and monocentric study, which included a limited number of patients. Second, a matched control group was not available. Third, SARS-CoV-2 variant typing was performed only in five patients of interest. Therefore, further studies are necessary to confirm the improvement of efficacy with combination therapy compared to monotherapy. Moreover, we were not able to assess from our study whether emergence of SARS-CoV-2 mutations was a direct consequence of monotherapy.

## Conclusions

In our patient group, a low rate of progression of COVID-19 requiring hospital admission was observed, which is in line with data reported in pivotal drug studies (0.7–6.8%) [12–17]. Since patients are carefully selected in randomized clinical trials, providing good adherence to study interventions, our real-life data are important to confirm the trial results. The dedicated COVID-19 proximity territorial service appeared to be able to strengthen the regional sanitary system, avoiding the overwhelming of other services. Moreover, our results appear to support early combination therapy as a possible strategy to improve the clinical outcome of immunocompromised patients, who display a high risk of clinical progression even when treated with monotherapy [51].

## Supplementary Information

**Additional file 1: Table S1. **Metrics of Next Generation Sequencing reported for each viral isolate.

**Additional file 2: Table S2.** Complete list of mutations identified in the SARS-CoV-2 genomes from patients described.

## Data Availability

The datasets used and/or analysed during the current study are de-identified and available from the corresponding author (torti@unicz.it) on reasonable request.

## Abbreviations

COVID-19: Coronavirus disease
SARS-CoV-2: Severe Acute Respiratory Syndrome Coronavirus-2
mAbs: Monoclonal antibodies
RMD: Remdesivir
MOL: Molnupiravir
NRM/r: Nirmatrelvir/ritonavir
BMI: Body mass index
WGS: Whole-genome sequencing
BAM/ETE: Bamlanivimab/etesevimab
SOT: Sotrovimab
NSTEMI: Non-ST-elevation myocardial infarction
CAS/IMD: Casirivimab/imdevimab
VOC: Variant of concern
CT: Computed tomography
