# Publisher Correction: Lessons learned and implications of early therapies for coronavirus disease in a territorial service centre in the Calabria region: a retrospective study

**DOI:** 10.1186/s12879-022-07871-9

**Published:** 2022-11-24

**Authors:** Vincenzo Scaglione, Salvatore Rotundo, Nadia Marascio, Carmela De Marco, Rosaria Lionello, Claudia Veneziano, Lavinia Berardelli, Angela Quirino, Vincenzo Olivadese, Francesca Serapide, Bruno Tassone, Helen Linda Morrone, Chiara Davoli, Valentina La Gamba, Andrea Bruni, Bruno Mario Cesana, Giovanni Matera, Alessandro Russo, Francesco Saverio Costanzo, Giuseppe Viglietto, Enrico Maria Trecarichi, Carlo Torti, IDTM U. M. G. COVID-19 Group, Enrico Maria Trecarichi, Alessandro Russo, Francesca Serapide, Bruno Tassone, Paolo Fusco, Vincenzo Scaglione, Chiara Davoli, Rosaria Lionello, Valentina La Gamba, Salvatore Rotundo, Helen Morrone, Lavinia Berardelli, Maria Teresa Tassone, Vincenzo Olivadese, Riccardo Serraino, Chiara Costa, Stefano Alcaro, Caterina De Filippo, Giovambattista De Sarro, Arturo Pujia, Aldo Quattrone, Francesco Saverio Costanzo, Giovanni Cuda, Daniela Patrizia Foti, Giuseppe Viglietto, Giovanni Matera, Federico Longhini, Andrea Bruni, Eugenio Garofalo, Eugenio Biamonte, Vincenzo Brescia, Domenico Laganà, Maria Petullà, Bernardo Bertucci, Angela Quirino, Giorgio Settimo Barreca, Aida Giancotti, Luigia Gallo, Angelo Lamberti, Nadia Marascio, Adele Emanuela De Francesco, Simona Mirarchi, Carlo Torti

**Affiliations:** 1 Chair of Infectious and Tropical Diseases, Department of Medical and Surgical Sciences, “Magna Græcia” University, Viale Europa, Loc. Germaneto, 88100 Catanzaro, Italy; 2 Chair of Clinical Microbiology, Department of Health Sciences, “Magna Græcia” University, Catanzaro, Italy; 3 Department of Experimental and Clinical Medicine, “Magna Græcia” University, Catanzaro, Italy; 4 Chair of Intensive Care, Department of Medical and Surgical Sciences, “Magna Græcia” University, Catanzaro, Italy; 5 Unit of Medical Statistics, Biometrics and Bioinformatics “Giulio A. Maccacaro”, Department of Clinical Sciences and Community Health, Faculty of Medicine and Surgery, University of Milan, Milan, Italy; 6 Department of Experimental and Clinical Medicine, Interdepartmental Center of Services (CIS), Molecular Genomics and Pathology, “Magna Græcia” University, Catanzaro, Italy

## Publisher Correction: BMC Infectious Diseases (2022) 22:793 https://doi.org/10.1186/s12879-022-07774-9

In the original publication of this article [1] the footnotes of Figure 1 were accidentally omitted during the publication process. In this correction article: Fig. 1 with the footnotes is published. The original article has been updated to rectify this error. The publisher apologizes to the authors and readers for the inconvenience caused.

**Fig. 1:**
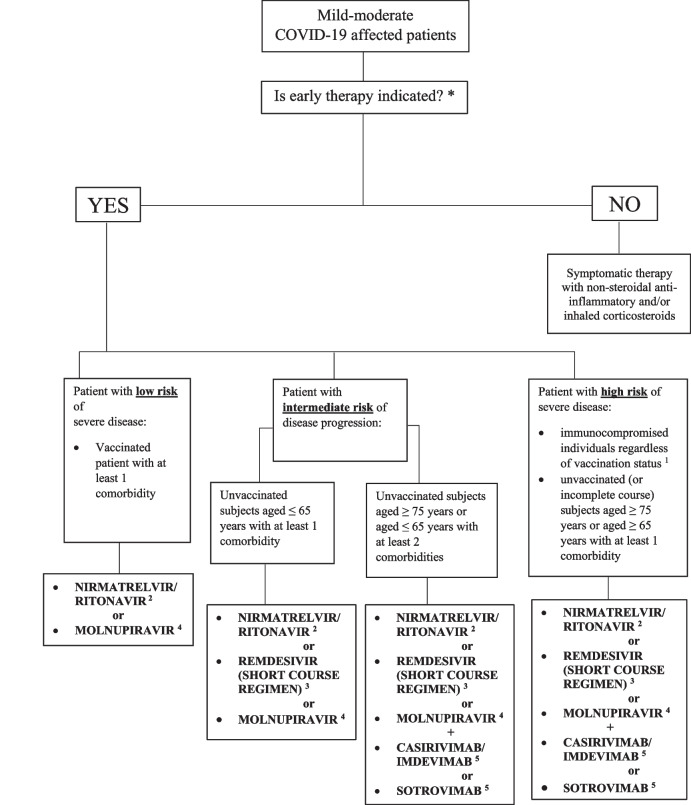
Flow chart of drugs prescription choices according to risk of progression of COVID-19. *Presence of at least one of the following factors: age > 65 years, BMI ≥ 30, patients chronically subjected to peritoneal dialysis or haemodialysis, uncontrolled diabetes mellitus or with chronic complications, primitive or secondary immunodeficiency (particularly concerning patients being treated with immunosuppressive drugs or less than 6 months from suspension of treatment), cardiocerebrovascular disease (including arterial hypertension with organ damage), COPD and/or other chronic respiratory diseases (lung fibrosis or patient needing O_2_-therapy for reasons different from SARS-CoV-2 infection), active oncological or oncohematological disease, chronic hepatopathy, hemoglobinopathies, neurodegenerative disorders. ^1^Patients affected by haematological malignancies/autoimmune diseases or treated with immunosuppressive drugs or transplant receivers; ^2^First choice in patients with eGFR > 30 ml/min and no major drug interactions; ^3^Useful in patients with eGFR > 30 ml/min if major drug interactions contraindicate nirmatrelvir/ritonavir or in patients with dysphagia; ^4^For use in patients with severe renal insufficiency and/or partially immunised (*i.e.*, previous SARS-CoV-2 infection, vaccination course incomplete or completed more than 6 months before); ^5^mAbs therapy was chosen considering local epidemiology of variants of concern
