# Supplementary material for: Lessons learned and implications of early therapies for coronavirus disease in a territorial service centre in the Calabria region: a retrospective study
Source: BMC Infect Dis. 2022 Oct 20;22:793. doi: 10.1186/s12879-022-07774-9 (PMC9583059; doi:10.1186/s12879-022-07774-9)
Supplement: Supplementary file 1 — Additional file 1: Table S1. Metrics of Next Generation Sequencing reported for each viral isolate. [file 12879_2022_7774_MOESM1_ESM.pdf]

Patient	Mapped Reads	Filtered Reads	Target Reads	Mean Depth	Uniformity
Patient1_030621	540222	0.03%	99.15%	2464	87.04%
Patient1_210621	411036	0.03%	96.18%	2115	86.17%
Patient1_060721	321277	0.00%	99.97%	2216	98.01%
Patient1_120721	1064840	0.68%	87.14%	5981	85.73%
Patient1_190721	365137	0.00%	99.97%	2538	99.08%
Patient1_260721	361195	0.00%	99.97%	2494	98.82%
Patient1_040821	609497	1.00%	89.04%	3665	97.30%
Patient2_210122	809219	0.00%	99.98%	5560	97.66%
Patient2_120222	745916	12.38%	89.82%	3988	97.33%
Patient3_060522	819621	0.00%	99.82%	5670	97.41%
Patient4_280322	720611	0.00%	99.95%	4944	97.58%
Patient5_090122	73123	0.02%	99.98%	549	86.16%

Supplementary Table 1: metrics of Next Generation Sequencing reported for each viral isolate.
