# Supplementary material for: Lessons learned and implications of early therapies for coronavirus disease in a territorial service centre in the Calabria region: a retrospective study
Source: BMC Infect Dis. 2022 Oct 20;22:793. doi: 10.1186/s12879-022-07774-9 (PMC9583059; doi:10.1186/s12879-022-07774-9)
Supplement: Supplementary file 2 — Additional file 2: Table S2. Complete list of mutations identified in the SARS-CoV-2 genomes from patients described. [file 12879_2022_7774_MOESM2_ESM.pdf]

Lineage	POS	REF	ALT	VARTYPE	GEN[*].GT	ANN[*].GENE	ANN[*].FEATUREID	EFF[*].RANK	ANN[*].HGVS_C	ANN[*].HGVS_P	ANN[*].EFFECT	EFF[*].IMPACT	AF	QUAL	DP	Isolate
B.1.1.7	241	C	T	SNP	Homozygous	orf1ab	QHD43415.1	-1	c.-25C>T	.	upstream_gene_variant	MODIFIER	1.0	2979.89	897	Patient1_030621
B.1.1.7	913	C	T	SNP	Homozygous	orf1ab	QHD43415.1	1	c.648C>T	p.Ser216Ser	synonymous_variant	LOW	1.0	2982.21	5925	Patient1_030621
B.1.1.7	3037	C	T	SNP	Homozygous	orf1ab	QHD43415.1	1	c.2772C>T	p.Phe924Phe	synonymous_variant	LOW	0.9975	2924.76	1360	Patient1_030621
B.1.1.7	3267	C	T	SNP	Homozygous	orf1ab	QHD43415.1	1	c.3002C>T	p.Thr1001Ile	missense_variant	MODERATE	1.0	1563.12	211	Patient1_030621
B.1.1.7	5388	C	A	SNP	Homozygous	orf1ab	QHD43415.1	1	c.5123C>A	p.Ala1708Asp	missense_variant	MODERATE	0.997487	2934.01	775	Patient1_030621
B.1.1.7	5986	C	T	SNP	Homozygous	orf1ab	QHD43415.1	1	c.5721C>T	p.Phe1907Phe	synonymous_variant	LOW	1.0	2657.5	354	Patient1_030621
B.1.1.7	6114	C	T	SNP	Homozygous	orf1ab	QHD43415.1	1	c.5849C>T	p.Pro1950Leu	missense_variant	MODERATE	0.995	2919.69	1284	Patient1_030621
B.1.1.7	6954	T	C	SNP	Homozygous	orf1ab	QHD43415.1	1	c.6689T>C	p.Ile2230Thr	missense_variant	MODERATE	0.933718	2073.43	347	Patient1_030621
B.1.1.7	7735	C	T	SNP	Homozygous	orf1ab	QHD43415.1	1	c.7470C>T	p.Ile2490Ile	synonymous_variant	LOW	1.0	2973.03	1164	Patient1_030621
B.1.1.7	11287	GTCTGGTTTT	G	DEL	Homozygous	orf1ab	QHD43415.1	1	c.11023_11031delTCTGGTTTT	p.Ser3675_Phe3677del	conservative_inframe_deletion	MODERATE	1.0	2980.37	1802	Patient1_030621
B.1.1.7	14408	C	T	SNP	Homozygous	orf1ab	QHD43415.1	2	c.14144C>T	p.Pro4715Leu	missense_variant	MODERATE	0.997118	2548.5	347	Patient1_030621
B.1.1.7	14676	C	T	SNP	Homozygous	orf1ab	QHD43415.1	2	c.14412C>T	p.Pro4804Pro	synonymous_variant	LOW	1.0	2553.92	343	Patient1_030621
B.1.1.7	15279	C	T	SNP	Homozygous	orf1ab	QHD43415.1	2	c.15015C>T	p.His5005His	synonymous_variant	LOW	1.0	2982.21	533	Patient1_030621
B.1.1.7	16176	T	C	SNP	Homozygous	orf1ab	QHD43415.1	2	c.15912T>C	p.Thr5304Thr	synonymous_variant	LOW	0.997494	2939.67	919	Patient1_030621
B.1.1.7	16391	C	T	SNP	Homozygous	orf1ab	QHD43415.1	2	c.16127C>T	p.Ala5376Val	missense_variant	MODERATE	0.9975	2948.9	1505	Patient1_030621
B.1.1.7	1761	A	G	SNP	Homozygou	orf1ab	QHD43415.1	2	c.17351A>G	p.Lys5784Arg	missense_variant	MODERATE	0.9975	2379.8	772	Patient1_0306

	5				s									2		21
B.1.1.7	21764	ATACATG	A	DEL	Homozygous	S	QHD43416.1	1	c.204_209delACATGT	p.His69_Val70del	disruptive_inframe_deletion	MODERATE	1.0	2982.21	461	Patient1_030621
B.1.1.7	21990	TTTA	T	DEL	Homozygous	S	QHD43416.1	1	c.432_434delTTA	p.Tyr145del	disruptive_inframe_deletion	MODERATE	1.0	2353.39	320	Patient1_030621
B.1.1.7	23063	A	T	SNP	Homozygous	S	QHD43416.1	1	c.1501A>T	p.Asn501Tyr	missense_variant	MODERATE	0.993333	869.016	150	Patient1_030621
B.1.1.7	23271	C	A	SNP	Homozygous	S	QHD43416.1	1	c.1709C>A	p.Ala570Asp	missense_variant	MODERATE	1.0	2967.33	4136	Patient1_030621
B.1.1.7	23403	A	G	SNP	Homozygous	S	QHD43416.1	1	c.1841A>G	p.Asp614Gly	missense_variant	MODERATE	1.0	2982.17	2661	Patient1_030621
B.1.1.7	23604	C	A	SNP	Homozygous	S	QHD43416.1	1	c.2042C>A	p.Pro681His	missense_variant	MODERATE	0.997475	2919.18	1223	Patient1_030621
B.1.1.7	23709	C	T	SNP	Homozygous	S	QHD43416.1	1	c.2147C>T	p.Thr716Ile	missense_variant	MODERATE	1.0	2982.12	742	Patient1_030621
B.1.1.7	24026	C	T	SNP	Homozygous	S	QHD43416.1	1	c.2464C>T	p.Leu822Phe	missense_variant	MODERATE	1.0	2940.79	482	Patient1_030621
B.1.1.7	24506	T	G	SNP	Homozygous	S	QHD43416.1	1	c.2944T>G	p.Ser982Ala	missense_variant	MODERATE	1.0	2982.04	2433	Patient1_030621
B.1.1.7	24914	G	C	SNP	Homozygous	S	QHD43416.1	1	c.3352G>C	p.Asp1118His	missense_variant	MODERATE	0.9975	2950.45	728	Patient1_030621
B.1.1.7	26730	G	C	SNP	Homozygous	M	QHD43419.1	1	c.208G>C	p.Val70Leu	missense_variant	MODERATE	1.0	2982.19	1295	Patient1_030621
B.1.1.7	27494	C	T	SNP	Homozygous	ORF7a	QHD43421.1	1	c.101C>T	p.Pro34Leu	missense_variant	MODERATE	0.9925	2891.92	526	Patient1_030621
B.1.1.7	27929	T	A	SNP	Homozygous	ORF8	QHD43422.1	1	c.36T>A	p.Thr12Thr	synonymous_variant	LOW	0.9325	2385.75	2137	Patient1_030621
B.1.1.7	27972	C	T	SNP	Homozygous	ORF8	QHD43422.1	1	c.79C>T	p.Gln27*	stop_gained	HIGH	0.985	2256.31	2481	Patient1_030621
B.1.1.7	28048	G	T	SNP	Homozygous	ORF8	QHD43422.1	1	c.155G>T	p.Arg52Ile	missense_variant	MODERATE	1.0	2971.27	3385	Patient1_030621
B.1.1.7	28111	A	G	SNP	Homozygous	ORF8	QHD43422.1	1	c.218A>G	p.Tyr73Cys	missense_variant	MODERATE	1.0	2982.21	1404	Patient1_030621

B.1.1.7	28270	TA	T	DEL	Homozygous	N	QHD43423.2	-1	c.-3delA	.	upstream_gene_variant	MODIFIER	1.0	2320.2	18945	Patient 1_030621
B.1.1.7	28280	GAT	CTA	MNP	Homozygous	N	QHD43423.2	1	c.7_9delGATinsCTA	p.Asp3Leu	missense_variant	MODERATE	1.0	2966.73	18848	Patient 1_030621
B.1.1.7	28881	GGG	AAC	MNP	Homozygous	N	QHD43423.2	1	c.608_610delGGGinsAAC	p.ArgGly203LysArg	missense_variant	MODERATE	1.0	2952.21	4077	Patient 1_030621
B.1.1.7	28977	C	T	SNP	Homozygous	N	QHD43423.2	1	c.704C>T	p.Ser235Phe	missense_variant	MODERATE	1.0	2981.98	3968	Patient 1_030621
B.1.1.7	29109	C	A	SNP	Homozygous	N	QHD43423.2	1	c.836C>A	p.Pro279Gln	missense_variant	MODERATE	0.9975	2379.9	2850	Patient 1_030621
B.1.1.7	241	C	T	SNP	Homozygous	orf1ab	QHD43415.1	-1	c.-25C>T	.	upstream_gene_variant	MODIFIER	1.0	1445.82	194	Patient 1_210621
B.1.1.7	913	C	T	SNP	Homozygous	orf1ab	QHD43415.1	1	c.648C>T	p.Ser216Ser	synonymous_variant	LOW	0.9975	2948.9	7405	Patient 1_210621
B.1.1.7	3037	C	T	SNP	Homozygous	orf1ab	QHD43415.1	1	c.2772C>T	p.Phe924Phe	synonymous_variant	LOW	1.0	2946.16	1904	Patient 1_210621
B.1.1.7	3267	C	T	SNP	Homozygous	orf1ab	QHD43415.1	1	c.3002C>T	p.Thr1001Ile	missense_variant	MODERATE	1.0	1946.73	261	Patient 1_210621
B.1.1.7	5388	C	A	SNP	Homozygous	orf1ab	QHD43415.1	1	c.5123C>A	p.Ala1708Asp	missense_variant	MODERATE	1.0	2982.19	786	Patient 1_210621
B.1.1.7	5986	C	T	SNP	Homozygous	orf1ab	QHD43415.1	1	c.5721C>T	p.Phe1907Phe	synonymous_variant	LOW	1.0	1290.68	172	Patient 1_210621
B.1.1.7	6114	C	T	SNP	Homozygous	orf1ab	QHD43415.1	1	c.5849C>T	p.Pro1950Leu	missense_variant	MODERATE	0.9925	2891.91	1094	Patient 1_210621
B.1.1.7	6954	T	C	SNP	Heterozygous	orf1ab	QHD43415.1	1	c.6689T>C	p.Ile2230Thr	missense_variant	MODERATE	0.817073	1038.83	246	Patient 1_210621
B.1.1.7	7735	C	T	SNP	Homozygous	orf1ab	QHD43415.1	1	c.7470C>T	p.Ile2490Ile	synonymous_variant	LOW	0.9975	2948.9	1022	Patient 1_210621
B.1.1.7	11287	GTCTGGTTTT	G	DEL	Homozygous	orf1ab	QHD43415.1	1	c.11023_11031delTCTGGTTTT	p.Ser3675_Phe3677del	conservative_inframe_deletion	MODERATE	1.0	2959.95	2246	Patient 1_210621
B.1.1.7	14408	C	T	SNP	Homozygous	orf1ab	QHD43415.1	2	c.14144C>T	p.Pro4715Leu	missense_variant	MODERATE	1.0	2045.11	275	Patient 1_210621
B.1.1.7	1467	C	T	SNP	Homozygou	orf1ab	QHD43415.1	2	c.14412C>T	p.Pro4804Pro	synonymous_variant	LOW	0.995	2913.3	493	Patient 1_2106

	6				s											21
B.1.1.7	15279	C	T	SNP	Homozygous	orf1ab	QHD43415.1	2	c.15015C>T	p.His5005His	synonymous_variant	LOW	1.0	2977.0	427	Patient 1_210621
B.1.1.7	16176	T	C	SNP	Homozygous	orf1ab	QHD43415.1	2	c.15912T>C	p.Thr5304Thr	synonymous_variant	LOW	1.0	2971.41	1659	Patient 1_210621
B.1.1.7	16391	C	T	SNP	Homozygous	orf1ab	QHD43415.1	2	c.16127C>T	p.Ala5376Val	missense_variant	MODERATE	0.997494	2939.98	2382	Patient 1_210621
B.1.1.7	17615	A	G	SNP	Homozygous	orf1ab	QHD43415.1	2	c.17351A>G	p.Lys5784Arg	missense_variant	MODERATE	0.995	2895.59	598	Patient 1_210621
B.1.1.7	21764	ATACATG	A	DEL	Homozygous	S	QHD43416.1	1	c.204_209delACATGT	p.His69_Val70del	disruptive_inframe_deletion	MODERATE	1.0	2133.31	287	Patient 1_210621
B.1.1.7	21990	TTTA	T	DEL	Homozygous	S	QHD43416.1	1	c.432_434delTTA	p.Tyr145del	disruptive_inframe_deletion	MODERATE	0.992366	2828.47	394	Patient 1_210621
B.1.1.7	23040	A	G	SNP	Homozygous	S	QHD43416.1	1	c.1478A>G	p.Gln493Arg	missense_variant	MODERATE	0.995	2919.64	774	Patient 1_210621
B.1.1.7	23063	A	T	SNP	Homozygous	S	QHD43416.1	1	c.1501A>T	p.Asn501Tyr	missense_variant	MODERATE	0.987879	1175.91	166	Patient 1_210621
B.1.1.7	23271	C	A	SNP	Homozygous	S	QHD43416.1	1	c.1709C>A	p.Ala570Asp	missense_variant	MODERATE	1.0	2974.33	2679	Patient 1_210621
B.1.1.7	23403	A	G	SNP	Homozygous	S	QHD43416.1	1	c.1841A>G	p.Asp614Gly	missense_variant	MODERATE	0.9975	2946.4	1936	Patient 1_210621
B.1.1.7	23604	C	A	SNP	Homozygous	S	QHD43416.1	1	c.2042C>A	p.Pro681His	missense_variant	MODERATE	1.0	2963.25	992	Patient 1_210621
B.1.1.7	23709	C	T	SNP	Homozygous	S	QHD43416.1	1	c.2147C>T	p.Thr716Ile	missense_variant	MODERATE	0.9975	2948.9	522	Patient 1_210621
B.1.1.7	24026	C	T	SNP	Homozygous	S	QHD43416.1	1	c.2464C>T	p.Leu822Phe	missense_variant	MODERATE	1.0	2960.28	752	Patient 1_210621
B.1.1.7	24506	T	G	SNP	Homozygous	S	QHD43416.1	1	c.2944T>G	p.Ser982Ala	missense_variant	MODERATE	1.0	2981.9	2819	Patient 1_210621
B.1.1.7	24914	G	C	SNP	Homozygous	S	QHD43416.1	1	c.3352G>C	p.Asp1118His	missense_variant	MODERATE	1.0	2974.75	465	Patient 1_210621
B.1.1.7	26730	G	C	SNP	Homozygous	M	QHD43419.1	1	c.208G>C	p.Val70Leu	missense_variant	MODERATE	1.0	2982.19	1818	Patient 1_210621

B.1.1.7	27494	C	T	SNP	Homozygous	ORF7a	QHD43421.1	1	c.101C>T	p.Pro34Leu	missense_variant	MODERATE	0.9825	2790.75	532	Patient 1_210621
B.1.1.7	27929	T	A	SNP	Homozygous	ORF8	QHD43422.1	1	c.36T>A	p.Thr12Thr	synonymous_variant	LOW	0.9599	2587.43	580	Patient 1_210621
B.1.1.7	27972	C	T	SNP	Homozygous	ORF8	QHD43422.1	1	c.79C>T	p.Gln27*	stop_gained	HIGH	1.0	2974.74	911	Patient 1_210621
B.1.1.7	28048	G	T	SNP	Homozygous	ORF8	QHD43422.1	1	c.155G>T	p.Arg52Ile	missense_variant	MODERATE	1.0	2982.21	1316	Patient 1_210621
B.1.1.7	28111	A	G	SNP	Homozygous	ORF8	QHD43422.1	1	c.218A>G	p.Tyr73Cys	missense_variant	MODERATE	1.0	2967.3	529	Patient 1_210621
B.1.1.7	28270	TA	T	DEL	Homozygous	N	QHD43423.2	-1	c.-3delA	.	upstream_gene_variant	MODIFIER	1.0	2369.69	10062	Patient 1_210621
B.1.1.7	28280	GAT	CTA	MNP	Homozygous	N	QHD43423.2	1	c.7_9delGATinsCTA	p.Asp3Leu	missense_variant	MODERATE	1.0	2967.62	9995	Patient 1_210621
B.1.1.7	28881	GGG	AAC	MNP	Homozygous	N	QHD43423.2	1	c.608_610delGGGinsAAC	p.ArgGly203LysArg	missense_variant	MODERATE	0.997475	2915.48	2026	Patient 1_210621
B.1.1.7	28977	C	T	SNP	Homozygous	N	QHD43423.2	1	c.704C>T	p.Ser235Phe	missense_variant	MODERATE	0.9975	2948.9	1999	Patient 1_210621
B.1.1.7	29109	C	A	SNP	Homozygous	N	QHD43423.2	1	c.836C>A	p.Pro279Gln	missense_variant	MODERATE	0.9925	2892.02	963	Patient 1_210621
B.1.1.7	241	C	T	SNP	Homozygous	orf1ab	QHD43415.1	-1	c.-25C>T	.	upstream_gene_variant	MODIFIER	0.9975	2936.6	990	Patient 1_060721
B.1.1.7	913	C	T	SNP	Homozygous	orf1ab	QHD43415.1	1	c.648C>T	p.Ser216Ser	synonymous_variant	LOW	0.9975	2948.91	4309	Patient 1_060721
B.1.1.7	3037	C	T	SNP	Homozygous	orf1ab	QHD43415.1	1	c.2772C>T	p.Phe924Phe	synonymous_variant	LOW	1.0	2949.14	2032	Patient 1_060721
B.1.1.7	3267	C	T	SNP	Homozygous	orf1ab	QHD43415.1	1	c.3002C>T	p.Thr1001Ile	missense_variant	MODERATE	1.0	2982.21	1349	Patient 1_060721
B.1.1.7	5388	C	A	SNP	Homozygous	orf1ab	QHD43415.1	1	c.5123C>A	p.Ala1708Asp	missense_variant	MODERATE	1.0	2944.17	2399	Patient 1_060721
B.1.1.7	5986	C	T	SNP	Homozygous	orf1ab	QHD43415.1	1	c.5721C>T	p.Phe1907Phe	synonymous_variant	LOW	1.0	2979.67	852	Patient 1_060721
B.1.1.7	6114	C	T	SNP	Homozygou	orf1ab	QHD43415.1	1	c.5849C>T	p.Pro1950Leu	missense_variant	MODERATE	0.9975	2361.0	2096	Patient 1_0607

					s									5		21
B.1.1.7	6954	T	C	SNP	Homozygous	orf1ab	QHD43415.1	1	c.6689T>C	p.Ile2230Thr	missense_variant	MODERATE	1.0	2982.2	890	Patient1_060721
B.1.1.7	7735	C	T	SNP	Homozygous	orf1ab	QHD43415.1	1	c.7470C>T	p.Ile2490Ile	synonymous_variant	LOW	1.0	2968.44	1677	Patient1_060721
B.1.1.7	11287	GTCTGGTTTT	G	DEL	Homozygous	orf1ab	QHD43415.1	1	c.11023_11031delTCTGGTTTT	p.Ser3675_Phe3677del	conservative_inframe_deletion	MODERATE	1.0	2958.33	2205	Patient1_060721
B.1.1.7	14408	C	T	SNP	Homozygous	orf1ab	QHD43415.1	2	c.14144C>T	p.Pro4715Leu	missense_variant	MODERATE	0.9975	2948.89	773	Patient1_060721
B.1.1.7	14676	C	T	SNP	Homozygous	orf1ab	QHD43415.1	2	c.14412C>T	p.Pro4804Pro	synonymous_variant	LOW	0.9975	2948.36	889	Patient1_060721
B.1.1.7	15279	C	T	SNP	Homozygous	orf1ab	QHD43415.1	2	c.15015C>T	p.His5005His	synonymous_variant	LOW	0.9975	2948.9	1321	Patient1_060721
B.1.1.7	16176	T	C	SNP	Homozygous	orf1ab	QHD43415.1	2	c.15912T>C	p.Thr5304Thr	synonymous_variant	LOW	0.9975	2379.88	2275	Patient1_060721
B.1.1.7	16391	C	T	SNP	Homozygous	orf1ab	QHD43415.1	2	c.16127C>T	p.Ala5376Val	missense_variant	MODERATE	1.0	2982.21	2764	Patient1_060721
B.1.1.7	17615	A	G	SNP	Homozygous	orf1ab	QHD43415.1	2	c.17351A>G	p.Lys5784Arg	missense_variant	MODERATE	0.9975	2379.9	2249	Patient1_060721
B.1.1.7	21764	ATACATG	A	DEL	Homozygous	S	QHD43416.1	1	c.204_209delACATGT	p.His69_Val70del	disruptive_inframe_deletion	MODERATE	1.0	2964.97	868	Patient1_060721
B.1.1.7	21990	TTTA	T	DEL	Homozygous	S	QHD43416.1	1	c.432_434delTTA	p.Tyr145del	disruptive_inframe_deletion	MODERATE	0.829843	2838.49	1316	Patient1_060721
B.1.1.7	22290	C	T	SNP	Heterozygous	S	QHD43416.1	1	c.728C>T	p.Ala243Val	missense_variant	MODERATE	0.6075	795.448	1511	Patient1_060721
B.1.1.7	23040	A	G	SNP	Homozygous	S	QHD43416.1	1	c.1478A>G	p.Gln493Arg	missense_variant	MODERATE	0.994987	2914.81	2182	Patient1_060721
B.1.1.7	23063	A	T	SNP	Homozygous	S	QHD43416.1	1	c.1501A>T	p.Asn501Tyr	missense_variant	MODERATE	0.98995	2852.26	718	Patient1_060721
B.1.1.7	23271	C	A	SNP	Homozygous	S	QHD43416.1	1	c.1709C>A	p.Ala570Asp	missense_variant	MODERATE	1.0	2982.2	3134	Patient1_060721
B.1.1.7	23403	A	G	SNP	Homozygous	S	QHD43416.1	1	c.1841A>G	p.Asp614Gly	missense_variant	MODERATE	0.994975	2906.64	3743	Patient1_060721

B.1.1.7	23604	C	A	SNP	Homozygous	S	QHD43416.1	1	c.2042C>A	p.Pro681His	missense_variant	MODERATE	1.0	2974.77	1674	Patient1_060721
B.1.1.7	23709	C	T	SNP	Homozygous	S	QHD43416.1	1	c.2147C>T	p.Thr716Ile	missense_variant	MODERATE	1.0	2982.18	1040	Patient1_060721
B.1.1.7	24026	C	T	SNP	Homozygous	S	QHD43416.1	1	c.2464C>T	p.Leu822Phe	missense_variant	MODERATE	0.9925	2838.94	1612	Patient1_060721
B.1.1.7	24374	C	T	SNP	Heterozygous	S	QHD43416.1	1	c.2812C>T	p.Leu938Phe	missense_variant	MODERATE	0.471178	399.479	736	Patient1_060721
B.1.1.7	24506	T	G	SNP	Homozygous	S	QHD43416.1	1	c.2944T>G	p.Ser982Ala	missense_variant	MODERATE	1.0	2982.0	2415	Patient1_060721
B.1.1.7	24914	G	C	SNP	Homozygous	S	QHD43416.1	1	c.3352G>C	p.Asp1118His	missense_variant	MODERATE	0.997494	2373.87	1372	Patient1_060721
B.1.1.7	26730	G	C	SNP	Homozygous	M	QHD43419.1	1	c.208G>C	p.Val70Leu	missense_variant	MODERATE	1.0	2982.16	2710	Patient1_060721
B.1.1.7	27494	C	T	SNP	Homozygous	ORF7a	QHD43421.1	1	c.101C>T	p.Pro34Leu	missense_variant	MODERATE	0.992481	2886.46	890	Patient1_060721
B.1.1.7	27929	T	A	SNP	Homozygous	ORF8	QHD43422.1	1	c.36T>A	p.Thr12Thr	synonymous_variant	LOW	1.0	2982.02	2361	Patient1_060721
B.1.1.7	27972	C	T	SNP	Homozygous	ORF8	QHD43422.1	1	c.79C>T	p.Gln27*	stop_gained	HIGH	0.9975	2948.9	5483	Patient1_060721
B.1.1.7	28048	G	T	SNP	Homozygous	ORF8	QHD43422.1	1	c.155G>T	p.Arg52Ile	missense_variant	MODERATE	1.0	2982.21	5484	Patient1_060721
B.1.1.7	28111	A	G	SNP	Homozygous	ORF8	QHD43422.1	1	c.218A>G	p.Tyr73Cys	missense_variant	MODERATE	1.0	2982.21	3140	Patient1_060721
B.1.1.7	28270	TA	T	DEL	Homozygous	N	QHD43423.2	-1	c.-3delA	.	upstream_gene_variant	MODIFIER	1.0	2340.34	7181	Patient1_060721
B.1.1.7	28280	GAT	CTA	MNP	Homozygous	N	QHD43423.2	1	c.7_9delGATinsCTA	p.Asp3Leu	missense_variant	MODERATE	1.0	2982.21	7153	Patient1_060721
B.1.1.7	28881	GGG	AAC	MNP	Homozygous	N	QHD43423.2	1	c.608_610delGGGinsAAC	p.ArgGly203LysArg	missense_variant	MODERATE	1.0	2982.21	2639	Patient1_060721
B.1.1.7	28977	C	T	SNP	Homozygous	N	QHD43423.2	1	c.704C>T	p.Ser235Phe	missense_variant	MODERATE	1.0	2981.67	2612	Patient1_060721
B.1.1.7	2910	C	A	SNP	Homozygou	N	QHD43423.2	1	c.836C>A	p.Pro279Gln	missense_variant	MODERATE	0.9974	2916.5	3035	Patient1_0607

	9				s								94			21
B.1.1.7	241	C	T	SNP	Homozygous	orf1ab	QHD43415.1	-1	c.-25C>T	.	upstream_gene_variant	MODIFIER	1.0	2974.47	1544	Patient1_120721
B.1.1.7	913	C	T	SNP	Homozygous	orf1ab	QHD43415.1	1	c.648C>T	p.Ser216Ser	synonymous_variant	LOW	0.9975	2948.83	9071	Patient1_120721
B.1.1.7	3037	C	T	SNP	Homozygous	orf1ab	QHD43415.1	1	c.2772C>T	p.Phe924Phe	synonymous_variant	LOW	1.0	2944.85	4884	Patient1_120721
B.1.1.7	3267	C	T	SNP	Homozygous	orf1ab	QHD43415.1	1	c.3002C>T	p.Thr1001Ile	missense_variant	MODERATE	0.9975	2951.18	1001	Patient1_120721
B.1.1.7	5388	C	A	SNP	Homozygous	orf1ab	QHD43415.1	1	c.5123C>A	p.Ala1708Asp	missense_variant	MODERATE	1.0	2974.67	2633	Patient1_120721
B.1.1.7	5986	C	T	SNP	Homozygous	orf1ab	QHD43415.1	1	c.5721C>T	p.Phe1907Phe	synonymous_variant	LOW	1.0	2979.43	664	Patient1_120721
B.1.1.7	6114	C	T	SNP	Homozygous	orf1ab	QHD43415.1	1	c.5849C>T	p.Pro1950Leu	missense_variant	MODERATE	0.997494	2942.67	2942	Patient1_120721
B.1.1.7	6954	T	C	SNP	Homozygous	orf1ab	QHD43415.1	1	c.6689T>C	p.Ile2230Thr	missense_variant	MODERATE	1.0	2982.21	529	Patient1_120721
B.1.1.7	7735	C	T	SNP	Homozygous	orf1ab	QHD43415.1	1	c.7470C>T	p.Ile2490Ile	synonymous_variant	LOW	1.0	2974.01	2044	Patient1_120721
B.1.1.7	11287	GTCTGGTTTT	G	DEL	Homozygous	orf1ab	QHD43415.1	1	c.11023_11031delTCTGGTTTT	p.Ser3675_Phe3677del	conservative_inframe_deletion	MODERATE	1.0	2952.02	4024	Patient1_120721
B.1.1.7	14408	C	T	SNP	Homozygous	orf1ab	QHD43415.1	2	c.14144C>T	p.Pro4715Leu	missense_variant	MODERATE	0.995	2914.35	1419	Patient1_120721
B.1.1.7	14676	C	T	SNP	Homozygous	orf1ab	QHD43415.1	2	c.14412C>T	p.Pro4804Pro	synonymous_variant	LOW	1.0	2944.87	1283	Patient1_120721
B.1.1.7	15279	C	T	SNP	Homozygous	orf1ab	QHD43415.1	2	c.15015C>T	p.His5005His	synonymous_variant	LOW	0.9975	2948.86	1403	Patient1_120721
B.1.1.7	16176	T	C	SNP	Homozygous	orf1ab	QHD43415.1	2	c.15912T>C	p.Thr5304Thr	synonymous_variant	LOW	0.995	2919.6	2072	Patient1_120721
B.1.1.7	16391	C	T	SNP	Homozygous	orf1ab	QHD43415.1	2	c.16127C>T	p.Ala5376Val	missense_variant	MODERATE	0.9975	2947.75	4291	Patient1_120721
B.1.1.7	17615	A	G	SNP	Homozygous	orf1ab	QHD43415.1	2	c.17351A>G	p.Lys5784Arg	missense_variant	MODERATE	0.992481	2885.05	3157	Patient1_120721

B.1.1.7	20104	C	T	SNP	Heterozygous	orf1ab	QHD43415.1	2	c.19840C>T	p.Leu6614Phe	missense_variant	MODERATE	0.274194	472.524	310	Patient1_120721
B.1.1.7	21764	ATACATG	A	DEL	Homozygous	S	QHD43416.1	1	c.204_209delACATGT	p.His69_Val70del	disruptive_inframe_deletion	MODERATE	1.0	2952.79	1992	Patient1_120721
B.1.1.7	21990	TTTA	T	DEL	Homozygous	S	QHD43416.1	1	c.432_434delTTA	p.Tyr145del	disruptive_inframe_deletion	MODERATE	1.0	2942.18	2778	Patient1_120721
B.1.1.7	22290	C	T	SNP	Heterozygous	S	QHD43416.1	1	c.728C>T	p.Ala243Val	missense_variant	MODERATE	0.2575	420.897	1978	Patient1_120721
B.1.1.7	22899	G	T	SNP	Heterozygous	S	QHD43416.1	1	c.1337G>T	p.Gly446Val	missense_variant	MODERATE	0.3175	108.227	9700	Patient1_120721
B.1.1.7	23040	A	G	SNP	Homozygous	S	QHD43416.1	1	c.1478A>G	p.Gln493Arg	missense_variant	MODERATE	1.0	2966.59	2821	Patient1_120721
B.1.1.7	23063	A	T	SNP	Homozygous	S	QHD43416.1	1	c.1501A>T	p.Asn501Tyr	missense_variant	MODERATE	1.0	2975.19	978	Patient1_120721
B.1.1.7	23271	C	A	SNP	Homozygous	S	QHD43416.1	1	c.1709C>A	p.Ala570Asp	missense_variant	MODERATE	1.0	2959.56	8374	Patient1_120721
B.1.1.7	23403	A	G	SNP	Homozygous	S	QHD43416.1	1	c.1841A>G	p.Asp614Gly	missense_variant	MODERATE	1.0	2973.76	9426	Patient1_120721
B.1.1.7	23604	C	A	SNP	Homozygous	S	QHD43416.1	1	c.2042C>A	p.Pro681His	missense_variant	MODERATE	1.0	2967.28	5064	Patient1_120721
B.1.1.7	23709	C	T	SNP	Homozygous	S	QHD43416.1	1	c.2147C>T	p.Thr716Ile	missense_variant	MODERATE	0.9925	2891.86	2433	Patient1_120721
B.1.1.7	24026	C	T	SNP	Homozygous	S	QHD43416.1	1	c.2464C>T	p.Leu822Phe	missense_variant	MODERATE	0.997462	2875.49	1426	Patient1_120721
B.1.1.7	24374	C	T	SNP	Heterozygous	S	QHD43416.1	1	c.2812C>T	p.Leu938Phe	missense_variant	MODERATE	0.375	195.857	1529	Patient1_120721
B.1.1.7	24506	T	G	SNP	Homozygous	S	QHD43416.1	1	c.2944T>G	p.Ser982Ala	missense_variant	MODERATE	0.9975	2948.77	6921	Patient1_120721
B.1.1.7	24914	G	C	SNP	Homozygous	S	QHD43416.1	1	c.3352G>C	p.Asp1118His	missense_variant	MODERATE	1.0	2959.96	3197	Patient1_120721
B.1.1.7	26730	G	C	SNP	Homozygous	M	QHD43419.1	1	c.208G>C	p.Val70Leu	missense_variant	MODERATE	1.0	2982.09	6709	Patient1_120721
B.1.1.7	2749	C	T	SNP	Homozygou	ORF7a	QHD43421.1	1	c.101C>T	p.Pro34Leu	missense_variant	MODERATE	0.9975	2948.8	3808	Patient1_1207

	4				s									6		21
B.1.1.7	27929	T	A	SNP	Homozygous	ORF8	QHD43422.1	1	c.36T>A	p.Thr12Thr	synonymous_variant	LOW	0.9975	2948.89	9288	Patient 1_120721
B.1.1.7	27972	C	T	SNP	Homozygous	ORF8	QHD43422.1	1	c.79C>T	p.Gln27*	stop_gained	HIGH	0.9975	2948.9	16706	Patient 1_120721
B.1.1.7	28048	G	T	SNP	Homozygous	ORF8	QHD43422.1	1	c.155G>T	p.Arg52Ile	missense_variant	MODERATE	1.0	2982.21	16879	Patient 1_120721
B.1.1.7	28111	A	G	SNP	Homozygous	ORF8	QHD43422.1	1	c.218A>G	p.Tyr73Cys	missense_variant	MODERATE	1.0	2963.02	7584	Patient 1_120721
B.1.1.7	28270	TA	T	DEL	Homozygous	N	QHD43423.2	-1	c.-3delA	.	upstream_gene_variant	MODIFIER	1.0	2310.97	61667	Patient 1_120721
B.1.1.7	28280	GAT	CTA	MNP	Homozygous	N	QHD43423.2	1	c.7_9delGATinsCTA	p.Asp3Leu	missense_variant	MODERATE	1.0	2959.79	61477	Patient 1_120721
B.1.1.7	28881	GGG	AAC	MNP	Homozygous	N	QHD43423.2	1	c.608_610delGGGinsAAC	p.ArgGly203LysArg	missense_variant	MODERATE	1.0	2967.34	25068	Patient 1_120721
B.1.1.7	28977	C	T	SNP	Homozygous	N	QHD43423.2	1	c.704C>T	p.Ser235Phe	missense_variant	MODERATE	0.995	2919.66	24962	Patient 1_120721
B.1.1.7	29109	C	A	SNP	Homozygous	N	QHD43423.2	1	c.836C>A	p.Pro279Gln	missense_variant	MODERATE	0.995	2919.82	15119	Patient 1_120721
B.1.1.7	241	C	T	SNP	Homozygous	orf1ab	QHD43415.1	-1	c.-25C>T	.	upstream_gene_variant	MODIFIER	0.9975	2942.59	1552	Patient 1_190721
B.1.1.7	913	C	T	SNP	Homozygous	orf1ab	QHD43415.1	1	c.648C>T	p.Ser216Ser	synonymous_variant	LOW	0.997494	2943.19	4162	Patient 1_190721
B.1.1.7	3037	C	T	SNP	Homozygous	orf1ab	QHD43415.1	1	c.2772C>T	p.Phe924Phe	synonymous_variant	LOW	1.0	2951.97	2600	Patient 1_190721
B.1.1.7	3267	C	T	SNP	Homozygous	orf1ab	QHD43415.1	1	c.3002C>T	p.Thr1001Ile	missense_variant	MODERATE	1.0	2982.2	1717	Patient 1_190721
B.1.1.7	5388	C	A	SNP	Homozygous	orf1ab	QHD43415.1	1	c.5123C>A	p.Ala1708Asp	missense_variant	MODERATE	1.0	2982.2	2143	Patient 1_190721
B.1.1.7	5986	C	T	SNP	Homozygous	orf1ab	QHD43415.1	1	c.5721C>T	p.Phe1907Phe	synonymous_variant	LOW	1.0	2974.3	977	Patient 1_190721
B.1.1.7	6114	C	T	SNP	Homozygous	orf1ab	QHD43415.1	1	c.5849C>T	p.Pro1950Leu	missense_variant	MODERATE	0.99	2865.75	2134	Patient 1_190721

B.1.1.7	6954	T	C	SNP	Homozygous	orf1ab	QHD43415.1	1	c.6689T>C	p.Ile2230Thr	missense_variant	MODERATE	0.997494	2941.47	1450	Patient1_190721
B.1.1.7	7735	C	T	SNP	Homozygous	orf1ab	QHD43415.1	1	c.7470C>T	p.Ile2490Ile	synonymous_variant	LOW	0.997494	2942.23	2401	Patient1_190721
B.1.1.7	11287	GTCTGGTTTT	G	DEL	Homozygous	orf1ab	QHD43415.1	1	c.11023_11031delTCTGGTTTT	p.Ser3675_Phe3677del	conservative_inframe_deletion	MODERATE	1.0	2975.32	2952	Patient1_190721
B.1.1.7	14408	C	T	SNP	Homozygous	orf1ab	QHD43415.1	2	c.14144C>T	p.Pro4715Leu	missense_variant	MODERATE	1.0	2982.2	1071	Patient1_190721
B.1.1.7	14676	C	T	SNP	Homozygous	orf1ab	QHD43415.1	2	c.14412C>T	p.Pro4804Pro	synonymous_variant	LOW	0.992481	2883.68	1401	Patient1_190721
B.1.1.7	15279	C	T	SNP	Homozygous	orf1ab	QHD43415.1	2	c.15015C>T	p.His5005His	synonymous_variant	LOW	1.0	2982.2	1615	Patient1_190721
B.1.1.7	16176	T	C	SNP	Homozygous	orf1ab	QHD43415.1	2	c.15912T>C	p.Thr5304Thr	synonymous_variant	LOW	0.99	2865.18	2808	Patient1_190721
B.1.1.7	16391	C	T	SNP	Homozygous	orf1ab	QHD43415.1	2	c.16127C>T	p.Ala5376Val	missense_variant	MODERATE	1.0	2982.12	3542	Patient1_190721
B.1.1.7	17615	A	G	SNP	Homozygous	orf1ab	QHD43415.1	2	c.17351A>G	p.Lys5784Arg	missense_variant	MODERATE	0.994987	2343.75	2379	Patient1_190721
B.1.1.7	20679	G	T	SNP	Heterozygous	orf1ab	QHD43415.1	2	c.20415G>T	p.Pro6805Pro	synonymous_variant	LOW	0.336683	135.002	1967	Patient1_190721
B.1.1.7	21764	ATACATG	A	DEL	Homozygous	S	QHD43416.1	1	c.204_209delACATGT	p.His69_Val70del	disruptive_inframe_deletion	MODERATE	1.0	2974.95	1919	Patient1_190721
B.1.1.7	21867	G	T	SNP	Heterozygous	S	QHD43416.1	1	c.305G>T	p.Arg102Ile	missense_variant	MODERATE	0.635	893.874	2495	Patient1_190721
B.1.1.7	21990	TTTA	T	DEL	Homozygous	S	QHD43416.1	1	c.432_434delTTA	p.Tyr145del	disruptive_inframe_deletion	MODERATE	1.0	2819.81	1495	Patient1_190721
B.1.1.7	22299	G	T	SNP	Heterozygous	S	QHD43416.1	1	c.737G>T	p.Arg246Ile	missense_variant	MODERATE	0.278195	620.671	2248	Patient1_190721
B.1.1.7	22899	G	T	SNP	Homozygous	S	QHD43416.1	1	c.1337G>T	p.Gly446Val	missense_variant	MODERATE	0.927318	2331.79	4382	Patient1_190721
B.1.1.7	23012	G	C	SNP	Heterozygous	S	QHD43416.1	1	c.1450G>C	p.Glu484Gln	missense_variant	MODERATE	0.6175	833.242	2837	Patient1_190721
B.1.1.7	2304	A	G	SNP	Homozygou	S	QHD43416.1	1	c.1478A>G	p.Gln493Arg	missense_variant	MODERATE	1.0	2939.0	2884	Patient1_1907

	0				s									9		21
B.1.1.7	23063	A	T	SNP	Homozygous	S	QHD43416.1	1	c.1501A>T	p.Asn501Tyr	missense_variant	MODERATE	1.0	2967.16	1039	Patient1_190721
B.1.1.7	23271	C	A	SNP	Homozygous	S	QHD43416.1	1	c.1709C>A	p.Ala570Asp	missense_variant	MODERATE	0.997494	2941.53	3062	Patient1_190721
B.1.1.7	23403	A	G	SNP	Homozygous	S	QHD43416.1	1	c.1841A>G	p.Asp614Gly	missense_variant	MODERATE	1.0	2981.89	3981	Patient1_190721
B.1.1.7	23604	C	A	SNP	Homozygous	S	QHD43416.1	1	c.2042C>A	p.Pro681His	missense_variant	MODERATE	1.0	2982.21	2299	Patient1_190721
B.1.1.7	23709	C	T	SNP	Homozygous	S	QHD43416.1	1	c.2147C>T	p.Thr716Ile	missense_variant	MODERATE	0.9975	2948.84	1477	Patient1_190721
B.1.1.7	24026	C	T	SNP	Homozygous	S	QHD43416.1	1	c.2464C>T	p.Leu822Phe	missense_variant	MODERATE	1.0	2913.2	1858	Patient1_190721
B.1.1.7	24506	T	G	SNP	Homozygous	S	QHD43416.1	1	c.2944T>G	p.Ser982Ala	missense_variant	MODERATE	1.0	2981.92	2640	Patient1_190721
B.1.1.7	24914	G	C	SNP	Homozygous	S	QHD43416.1	1	c.3352G>C	p.Asp1118His	missense_variant	MODERATE	0.9975	2948.87	2317	Patient1_190721
B.1.1.7	26428	G	T	SNP	Heterozygous	E	QHD43418.1	1	c.184G>T	p.Val62Phe	missense_variant	MODERATE	0.2825	667.354	2114	Patient1_190721
B.1.1.7	26730	G	C	SNP	Homozygous	M	QHD43419.1	1	c.208G>C	p.Val70Leu	missense_variant	MODERATE	1.0	2982.2	2450	Patient1_190721
B.1.1.7	27494	C	T	SNP	Homozygous	ORF7a	QHD43421.1	1	c.101C>T	p.Pro34Leu	missense_variant	MODERATE	1.0	2954.81	1684	Patient1_190721
B.1.1.7	27929	T	A	SNP	Homozygous	ORF8	QHD43422.1	1	c.36T>A	p.Thr12Thr	synonymous_variant	LOW	1.0	2981.68	3745	Patient1_190721
B.1.1.7	27972	C	T	SNP	Homozygous	ORF8	QHD43422.1	1	c.79C>T	p.Gln27*	stop_gained	HIGH	0.9975	2948.75	9146	Patient1_190721
B.1.1.7	28048	G	T	SNP	Homozygous	ORF8	QHD43422.1	1	c.155G>T	p.Arg52Ile	missense_variant	MODERATE	1.0	2974.77	9188	Patient1_190721
B.1.1.7	28111	A	G	SNP	Homozygous	ORF8	QHD43422.1	1	c.218A>G	p.Tyr73Cys	missense_variant	MODERATE	0.9975	2949.83	5418	Patient1_190721
B.1.1.7	28270	TA	T	DEL	Homozygous	N	QHD43423.2	-1	c.-3delA	.	upstream_gene_variant	MODIFIER	1.0	2346.24	9698	Patient1_190721

B.1.1.7	28280	GAT	CTA	MNP	Homozygous	N	QHD43423.2	1	c.7_9delGATinsCTA	p.Asp3Leu	missense_variant	MODERATE	1.0	2967.16	9661	Patient 1_190721
B.1.1.7	28881	GGG	AAC	MNP	Homozygous	N	QHD43423.2	1	c.608_610delGGGinsAAC	p.ArgGly203LysArg	missense_variant	MODERATE	1.0	2974.77	4018	Patient 1_190721
B.1.1.7	28977	C	T	SNP	Homozygous	N	QHD43423.2	1	c.704C>T	p.Ser235Phe	missense_variant	MODERATE	0.9975	2948.9	4008	Patient 1_190721
B.1.1.7	29109	C	A	SNP	Homozygous	N	QHD43423.2	1	c.836C>A	p.Pro279Gln	missense_variant	MODERATE	1.0	2982.21	5013	Patient 1_190721
B.1.1.7	241	C	T	SNP	Homozygous	orf1ab	QHD43415.1	-1	c.-25C>T	.	upstream_gene_variant	MODIFIER	1.0	2965.39	1228	Patient 1_260721
B.1.1.7	913	C	T	SNP	Homozygous	orf1ab	QHD43415.1	1	c.648C>T	p.Ser216Ser	synonymous_variant	LOW	0.995	2353.44	4524	Patient 1_260721
B.1.1.7	3037	C	T	SNP	Homozygous	orf1ab	QHD43415.1	1	c.2772C>T	p.Phe924Phe	synonymous_variant	LOW	1.0	2927.91	2610	Patient 1_260721
B.1.1.7	3267	C	T	SNP	Homozygous	orf1ab	QHD43415.1	1	c.3002C>T	p.Thr1001Ile	missense_variant	MODERATE	0.9975	2948.84	1580	Patient 1_260721
B.1.1.7	5388	C	A	SNP	Homozygous	orf1ab	QHD43415.1	1	c.5123C>A	p.Ala1708Asp	missense_variant	MODERATE	1.0	2982.18	2759	Patient 1_260721
B.1.1.7	5986	C	T	SNP	Homozygous	orf1ab	QHD43415.1	1	c.5721C>T	p.Phe1907Phe	synonymous_variant	LOW	1.0	2967.32	897	Patient 1_260721
B.1.1.7	6114	C	T	SNP	Homozygous	orf1ab	QHD43415.1	1	c.5849C>T	p.Pro1950Leu	missense_variant	MODERATE	0.995	2919.64	2268	Patient 1_260721
B.1.1.7	6954	T	C	SNP	Homozygous	orf1ab	QHD43415.1	1	c.6689T>C	p.Ile2230Thr	missense_variant	MODERATE	1.0	2982.21	1338	Patient 1_260721
B.1.1.7	7735	C	T	SNP	Homozygous	orf1ab	QHD43415.1	1	c.7470C>T	p.Ile2490Ile	synonymous_variant	LOW	0.9975	2947.92	2045	Patient 1_260721
B.1.1.7	11287	GTCTGGTTTT	G	DEL	Homozygous	orf1ab	QHD43415.1	1	c.11023_11031delTCTGGTTTT	p.Ser3675_Phe3677del	conservative_inframe_deletion	MODERATE	1.0	2871.08	2651	Patient 1_260721
B.1.1.7	14408	C	T	SNP	Homozygous	orf1ab	QHD43415.1	2	c.14144C>T	p.Pro4715Leu	missense_variant	MODERATE	0.9975	2945.06	1107	Patient 1_260721
B.1.1.7	14676	C	T	SNP	Homozygous	orf1ab	QHD43415.1	2	c.14412C>T	p.Pro4804Pro	synonymous_variant	LOW	0.997494	2941.46	1226	Patient 1_260721
B.1.1.7	1527	C	T	SNP	Homozygou	orf1ab	QHD43415.1	2	c.15015C>T	p.His5005His	synonymous_variant	LOW	0.9975	2948.9	1189	Patient 1_2607

	9				s									1		21
B.1.1.7	16176	T	C	SNP	Homozygous	orf1ab	QHD43415.1	2	c.15912T>C	p.Thr5304Thr	synonymous_variant	LOW	0.995	2919.58	2784	Patient1_260721
B.1.1.7	16391	C	T	SNP	Homozygous	orf1ab	QHD43415.1	2	c.16127C>T	p.Ala5376Val	missense_variant	MODERATE	0.989975	2857.38	3190	Patient1_260721
B.1.1.7	17615	A	G	SNP	Homozygous	orf1ab	QHD43415.1	2	c.17351A>G	p.Lys5784Arg	missense_variant	MODERATE	0.9975	2365.43	2468	Patient1_260721
B.1.1.7	21764	ATACATG	A	DEL	Homozygous	S	QHD43416.1	1	c.204_209delACATGT	p.His69_Val70del	disruptive_inframe_deletion	MODERATE	1.0	2974.77	1350	Patient1_260721
B.1.1.7	21867	G	T	SNP	Homozygous	S	QHD43416.1	1	c.305G>T	p.Arg102Ile	missense_variant	MODERATE	1.0	2982.13	2225	Patient1_260721
B.1.1.7	21990	TTTA	T	DEL	Homozygous	S	QHD43416.1	1	c.432_434delTTA	p.Tyr145del	disruptive_inframe_deletion	MODERATE	0.997468	2906.07	1438	Patient1_260721
B.1.1.7	22205	G	C	SNP	Homozygous	S	QHD43416.1	1	c.643G>C	p.Asp215His	missense_variant	MODERATE	0.969925	2670.75	1083	Patient1_260721
B.1.1.7	22899	G	T	SNP	Homozygous	S	QHD43416.1	1	c.1337G>T	p.Gly446Val	missense_variant	MODERATE	0.967419	2635.56	4839	Patient1_260721
B.1.1.7	23012	G	C	SNP	Homozygous	S	QHD43416.1	1	c.1450G>C	p.Glu484Gln	missense_variant	MODERATE	1.0	2982.21	2546	Patient1_260721
B.1.1.7	23040	A	G	SNP	Homozygous	S	QHD43416.1	1	c.1478A>G	p.Gln493Arg	missense_variant	MODERATE	0.992481	2885.09	2604	Patient1_260721
B.1.1.7	23063	A	T	SNP	Homozygous	S	QHD43416.1	1	c.1501A>T	p.Asn501Tyr	missense_variant	MODERATE	0.9975	2948.11	787	Patient1_260721
B.1.1.7	23271	C	A	SNP	Homozygous	S	QHD43416.1	1	c.1709C>A	p.Ala570Asp	missense_variant	MODERATE	0.9975	2948.79	2908	Patient1_260721
B.1.1.7	23403	A	G	SNP	Homozygous	S	QHD43416.1	1	c.1841A>G	p.Asp614Gly	missense_variant	MODERATE	1.0	2978.62	3855	Patient1_260721
B.1.1.7	23604	C	A	SNP	Homozygous	S	QHD43416.1	1	c.2042C>A	p.Pro681His	missense_variant	MODERATE	0.997494	2374.12	1929	Patient1_260721
B.1.1.7	23709	C	T	SNP	Homozygous	S	QHD43416.1	1	c.2147C>T	p.Thr716Ile	missense_variant	MODERATE	1.0	2964.14	1257	Patient1_260721
B.1.1.7	24026	C	T	SNP	Homozygous	S	QHD43416.1	1	c.2464C>T	p.Leu822Phe	missense_variant	MODERATE	0.994975	2879.74	1909	Patient1_260721

B.1.1.7	24506	T	G	SNP	Homozygous	S	QHD43416.1	1	c.2944T>G	p.Ser982Ala	missense_variant	MODERATE	1.0	2974.44	2871	Patient 1_260721
B.1.1.7	24914	G	C	SNP	Homozygous	S	QHD43416.1	1	c.3352G>C	p.Asp1118His	missense_variant	MODERATE	1.0	2982.19	1883	Patient 1_260721
B.1.1.7	26730	G	C	SNP	Homozygous	M	QHD43419.1	1	c.208G>C	p.Val70Leu	missense_variant	MODERATE	1.0	2982.19	2935	Patient 1_260721
B.1.1.7	27494	C	T	SNP	Homozygous	ORF7a	QHD43421.1	1	c.101C>T	p.Pro34Leu	missense_variant	MODERATE	0.9975	2364.42	1251	Patient 1_260721
B.1.1.7	27929	T	A	SNP	Homozygous	ORF8	QHD43422.1	1	c.36T>A	p.Thr12Thr	synonymous_variant	LOW	1.0	2982.21	2531	Patient 1_260721
B.1.1.7	27972	C	T	SNP	Homozygous	ORF8	QHD43422.1	1	c.79C>T	p.Gln27*	stop_gained	HIGH	0.9975	2948.9	5891	Patient 1_260721
B.1.1.7	28048	G	T	SNP	Homozygous	ORF8	QHD43422.1	1	c.155G>T	p.Arg52Ile	missense_variant	MODERATE	1.0	2982.19	5917	Patient 1_260721
B.1.1.7	28111	A	G	SNP	Homozygous	ORF8	QHD43422.1	1	c.218A>G	p.Tyr73Cys	missense_variant	MODERATE	1.0	2982.21	3380	Patient 1_260721
B.1.1.7	28270	TA	T	DEL	Homozygous	N	QHD43423.2	-1	c.-3delA	.	upstream_gene_variant	MODIFIER	1.0	2349.91	7835	Patient 1_260721
B.1.1.7	28280	GAT	CTA	MNP	Homozygous	N	QHD43423.2	1	c.7_9delGATinsCTA	p.Asp3Leu	missense_variant	MODERATE	1.0	2932.0	7802	Patient 1_260721
B.1.1.7	28881	GGG	AAC	MNP	Homozygous	N	QHD43423.2	1	c.608_610delGGGinsAAC	p.ArgGly203LysArg	missense_variant	MODERATE	1.0	2974.77	3152	Patient 1_260721
B.1.1.7	28977	C	T	SNP	Homozygous	N	QHD43423.2	1	c.704C>T	p.Ser235Phe	missense_variant	MODERATE	0.997487	2935.85	3127	Patient 1_260721
B.1.1.7	29109	C	A	SNP	Homozygous	N	QHD43423.2	1	c.836C>A	p.Pro279Gln	missense_variant	MODERATE	1.0	2982.2	3558	Patient 1_260721
B.1.1.7	241	C	T	SNP	Homozygous	orf1ab	QHD43415.1	-1	c.-25C>T	.	upstream_gene_variant	MODIFIER	1.0	2971.24	1488	Patient 1_040821
B.1.1.7	913	C	T	SNP	Homozygous	orf1ab	QHD43415.1	1	c.648C>T	p.Ser216Ser	synonymous_variant	LOW	0.9975	2380.02	7999	Patient 1_040821
B.1.1.7	3037	C	T	SNP	Homozygous	orf1ab	QHD43415.1	1	c.2772C>T	p.Phe924Phe	synonymous_variant	LOW	1.0	2952.03	3473	Patient 1_040821
B.1.1.7	3267	C	T	SNP	Homozygou	orf1ab	QHD43415.1	1	c.3002C>T	p.Thr1001Ile	missense_variant	MODERATE	1.0	2982.2	1847	Patient 1_0408

					s										21
B.1.1.7	5388	C	A	SNP	Homozygous	orf1ab	QHD43415.1	1	c.5123C>A	p.Ala1708Asp	missense_variant	MODERATE	1.0	2974.75	4968	Patient 1_040821
B.1.1.7	5986	C	T	SNP	Homozygous	orf1ab	QHD43415.1	1	c.5721C>T	p.Phe1907Phe	synonymous_variant	LOW	1.0	2962.54	1154	Patient 1_040821
B.1.1.7	6114	C	T	SNP	Homozygous	orf1ab	QHD43415.1	1	c.5849C>T	p.Pro1950Leu	missense_variant	MODERATE	0.995	2920.13	4425	Patient 1_040821
B.1.1.7	6954	T	C	SNP	Homozygous	orf1ab	QHD43415.1	1	c.6689T>C	p.Ile2230Thr	missense_variant	MODERATE	1.0	2958.19	1896	Patient 1_040821
B.1.1.7	7735	C	T	SNP	Homozygous	orf1ab	QHD43415.1	1	c.7470C>T	p.Ile2490Ile	synonymous_variant	LOW	0.9975	2948.7	3232	Patient 1_040821
B.1.1.7	11287	GTCTGGTTTT	G	DEL	Homozygous	orf1ab	QHD43415.1	1	c.11023_11031delTCTGGTTTT	p.Ser3675_Phe3677del	conservative_inframe_deletion	MODERATE	1.0	2936.84	3856	Patient 1_040821
B.1.1.7	14408	C	T	SNP	Homozygous	orf1ab	QHD43415.1	2	c.14144C>T	p.Pro4715Leu	missense_variant	MODERATE	0.989924	2840.41	1179	Patient 1_040821
B.1.1.7	14676	C	T	SNP	Homozygous	orf1ab	QHD43415.1	2	c.14412C>T	p.Pro4804Pro	synonymous_variant	LOW	0.9975	2946.07	1663	Patient 1_040821
B.1.1.7	15279	C	T	SNP	Homozygous	orf1ab	QHD43415.1	2	c.15015C>T	p.His5005His	synonymous_variant	LOW	0.995	2919.53	2810	Patient 1_040821
B.1.1.7	16176	T	C	SNP	Homozygous	orf1ab	QHD43415.1	2	c.15912T>C	p.Thr5304Thr	synonymous_variant	LOW	0.99	2865.23	4139	Patient 1_040821
B.1.1.7	16391	C	T	SNP	Homozygous	orf1ab	QHD43415.1	2	c.16127C>T	p.Ala5376Val	missense_variant	MODERATE	1.0	2982.2	5174	Patient 1_040821
B.1.1.7	17615	A	G	SNP	Homozygous	orf1ab	QHD43415.1	2	c.17351A>G	p.Lys5784Arg	missense_variant	MODERATE	1.0	2939.73	3913	Patient 1_040821
B.1.1.7	21761	G	T	SNP	Heterozygous	S	QHD43416.1	1	c.199G>T	p.Ala67Ser	missense_variant	MODERATE	0.2125	135.245	782	Patient 1_040821
B.1.1.7	21764	ATACATG	A	DEL	Homozygous	S	QHD43416.1	1	c.204_209delACATGT	p.His69_Val70del	disruptive_inframe_deletion	MODERATE	1.0	2945.16	781	Patient 1_040821
B.1.1.7	21867	G	T	SNP	Homozygous	S	QHD43416.1	1	c.305G>T	p.Arg102Ile	missense_variant	MODERATE	0.992481	2884.93	2634	Patient 1_040821
B.1.1.7	21990	TTTA	T	DEL	Homozygous	S	QHD43416.1	1	c.432_434delTTA	p.Tyr145del	disruptive_inframe_deletion	MODERATE	1.0	2964.73	2067	Patient 1_040821

B.1.1.7	22205	G	C	SNP	Homozygous	S	QHD43416.1	1	c.643G>C	p.Asp215His	missense_variant	MODERATE	1.0	2982.14	1178	Patient 1_040821
B.1.1.7	22317	G	T	SNP	Heterozygous	S	QHD43416.1	1	c.755G>T	p.Gly252Val	missense_variant	MODERATE	0.6925	1105.79	2183	Patient 1_040821
B.1.1.7	22899	G	T	SNP	Homozygous	S	QHD43416.1	1	c.1337G>T	p.Gly446Val	missense_variant	MODERATE	0.977444	2722.47	6524	Patient 1_040821
B.1.1.7	23012	G	C	SNP	Homozygous	S	QHD43416.1	1	c.1450G>C	p.Glu484Gln	missense_variant	MODERATE	0.997487	2934.09	3161	Patient 1_040821
B.1.1.7	23040	A	G	SNP	Homozygous	S	QHD43416.1	1	c.1478A>G	p.Gln493Arg	missense_variant	MODERATE	0.9975	2948.89	3215	Patient 1_040821
B.1.1.7	23063	A	T	SNP	Homozygous	S	QHD43416.1	1	c.1501A>T	p.Asn501Tyr	missense_variant	MODERATE	0.995	2919.37	1020	Patient 1_040821
B.1.1.7	23271	C	A	SNP	Homozygous	S	QHD43416.1	1	c.1709C>A	p.Ala570Asp	missense_variant	MODERATE	0.9975	2948.88	5010	Patient 1_040821
B.1.1.7	23280	C	T	SNP	Heterozygous	S	QHD43416.1	1	c.1718C>T	p.Thr573Ile	missense_variant	MODERATE	0.2625	46.16	5083	Patient 1_040821
B.1.1.7	23403	A	G	SNP	Homozygous	S	QHD43416.1	1	c.1841A>G	p.Asp614Gly	missense_variant	MODERATE	1.0	2976.78	5836	Patient 1_040821
B.1.1.7	23604	C	A	SNP	Homozygous	S	QHD43416.1	1	c.2042C>A	p.Pro681His	missense_variant	MODERATE	1.0	2982.21	2914	Patient 1_040821
B.1.1.7	23709	C	T	SNP	Homozygous	S	QHD43416.1	1	c.2147C>T	p.Thr716Ile	missense_variant	MODERATE	1.0	2981.71	1552	Patient 1_040821
B.1.1.7	24026	C	T	SNP	Homozygous	S	QHD43416.1	1	c.2464C>T	p.Leu822Phe	missense_variant	MODERATE	0.997481	2899.45	3312	Patient 1_040821
B.1.1.7	24506	T	G	SNP	Homozygous	S	QHD43416.1	1	c.2944T>G	p.Ser982Ala	missense_variant	MODERATE	0.997494	2942.53	3756	Patient 1_040821
B.1.1.7	24914	G	C	SNP	Homozygous	S	QHD43416.1	1	c.3352G>C	p.Asp1118His	missense_variant	MODERATE	0.9975	2948.93	1874	Patient 1_040821
B.1.1.7	26730	G	C	SNP	Homozygous	M	QHD43419.1	1	c.208G>C	p.Val70Leu	missense_variant	MODERATE	1.0	2982.18	3763	Patient 1_040821
B.1.1.7	27494	C	T	SNP	Homozygous	ORF7a	QHD43421.1	1	c.101C>T	p.Pro34Leu	missense_variant	MODERATE	0.99	2865.56	1156	Patient 1_040821
B.1.1.7	2792	T	A	SNP	Homozygou	ORF8	QHD43422.1	1	c.36T>A	p.Thr12Thr	synonymous_variant	LOW	0.9975	2948.9	3539	Patient 1_0408

	9				s											21
B.1.1.7	27972	C	T	SNP	Homozygous	ORF8	QHD43422.1	1	c.79C>T	p.Gln27*	stop_gained	HIGH	1.0	2960.04	7416	Patient 1_040821
B.1.1.7	28048	G	T	SNP	Homozygous	ORF8	QHD43422.1	1	c.155G>T	p.Arg52Ile	missense_variant	MODERATE	0.995	2919.85	7459	Patient 1_040821
B.1.1.7	28111	A	G	SNP	Homozygous	ORF8	QHD43422.1	1	c.218A>G	p.Tyr73Cys	missense_variant	MODERATE	0.995	2919.66	3908	Patient 1_040821
B.1.1.7	28270	TA	T	DEL	Homozygous	N	QHD43423.2	-1	c.-3delA	.	upstream_gene_variant	MODIFIER	1.0	2267.41	9230	Patient 1_040821
B.1.1.7	28280	GAT	CTA	MNP	Homozygous	N	QHD43423.2	1	c.7_9delGATinsCTA	p.Asp3Leu	missense_variant	MODERATE	1.0	2964.78	9197	Patient 1_040821
B.1.1.7	28881	GGG	AAC	MNP	Homozygous	N	QHD43423.2	1	c.608_610delGGGinsAAC	p.ArgGly203LysArg	missense_variant	MODERATE	1.0	2934.12	3696	Patient 1_040821
B.1.1.7	28977	C	T	SNP	Homozygous	N	QHD43423.2	1	c.704C>T	p.Ser235Phe	missense_variant	MODERATE	1.0	2982.21	3679	Patient 1_040821
B.1.1.7	29109	C	A	SNP	Homozygous	N	QHD43423.2	1	c.836C>A	p.Pro279Gln	missense_variant	MODERATE	1.0	2982.2	2840	Patient 1_040821
B.1.1.529 (BA.1.17)	241	C	T	SNP	Homozygous	orf1ab	QHD43415.1	-1	c.-25C>T	.	upstream_gene_variant	MODIFIER	1.0	2976.2	3131	Patient 2_210122
B.1.1.529 (BA.1.17)	2832	A	G	SNP	Homozygous	orf1ab	QHD43415.1	1	c.2567A>G	p.Lys856Arg	missense_variant	MODERATE	0.9975	2948.65	7046	Patient 2_210122
B.1.1.529 (BA.1.17)	3037	C	T	SNP	Homozygous	orf1ab	QHD43415.1	1	c.2772C>T	p.Phe924Phe	synonymous_variant	LOW	1.0	2947.12	7911	Patient 2_210122
B.1.1.529 (BA.1.17)	5386	T	G	SNP	Homozygous	orf1ab	QHD43415.1	1	c.5121T>G	p.Ala1707Ala	synonymous_variant	LOW	0.9975	2948.88	5069	Patient 2_210122
B.1.1.529 (BA.1.17)	5672	C	T	SNP	Homozygous	orf1ab	QHD43415.1	1	c.5407C>T	p.Pro1803Ser	missense_variant	MODERATE	0.995	2919.66	3292	Patient 2_210122
B.1.1.529 (BA.1.17)	5924	G	A	SNP	Homozygous	orf1ab	QHD43415.1	1	c.5659G>A	p.Val1887Ile	missense_variant	MODERATE	0.994987	2912.06	4995	Patient 2_210122

B.1.1.529 (BA.1.17)	6512	AGTT	A	DEL	Homozygous	orf1ab	QHD43415.1	1	c.6248_6250delGTT	p.Ser2083_Leu2084delinsIle	disruptive_inframe_deletion	MODERATE	1.0	2956.94	4309	Patient2_210122
B.1.1.529 (BA.1.17)	8393	G	A	SNP	Homozygous	orf1ab	QHD43415.1	1	c.8128G>A	p.Ala2710Thr	missense_variant	MODERATE	1.0	2982.21	5441	Patient2_210122
B.1.1.529 (BA.1.17)	10029	C	T	SNP	Homozygous	orf1ab	QHD43415.1	1	c.9764C>T	p.Thr3255Ile	missense_variant	MODERATE	1.0	2982.2	6038	Patient2_210122
B.1.1.529 (BA.1.17)	10449	C	A	SNP	Homozygous	orf1ab	QHD43415.1	1	c.10184C>A	p.Pro3395His	missense_variant	MODERATE	0.995	2354.22	3438	Patient2_210122
B.1.1.529 (BA.1.17)	11282	AGTTTGTCTG	A	DEL	Homozygous	orf1ab	QHD43415.1	1	c.11022_11030delGTCTGGTTT	p.Leu3674_Gly3676del	disruptive_inframe_deletion	MODERATE	1.0	2935.03	7161	Patient2_210122
B.1.1.529 (BA.1.17)	11537	A	G	SNP	Homozygous	orf1ab	QHD43415.1	1	c.11272A>G	p.Ile3758Val	missense_variant	MODERATE	1.0	2982.1	4840	Patient2_210122
B.1.1.529 (BA.1.17)	13195	T	C	SNP	Homozygous	orf1ab	QHD43415.1	1	c.12930T>C	p.Val4310Val	synonymous_variant	LOW	1.0	2982.18	5986	Patient2_210122
B.1.1.529 (BA.1.17)	14408	C	T	SNP	Homozygous	orf1ab	QHD43415.1	2	c.14144C>T	p.Pro4715Leu	missense_variant	MODERATE	1.0	2977.38	2475	Patient2_210122
B.1.1.529 (BA.1.17)	15240	C	T	SNP	Homozygous	orf1ab	QHD43415.1	2	c.14976C>T	p.Asn4992Asn	synonymous_variant	LOW	1.0	2976.85	5216	Patient2_210122
B.1.1.529 (BA.1.17)	18163	A	G	SNP	Homozygous	orf1ab	QHD43415.1	2	c.17899A>G	p.Ile5967Val	missense_variant	MODERATE	1.0	2982.16	3049	Patient2_210122
B.1.1.529 (BA.1.17)	19374	C	T	SNP	Homozygous	orf1ab	QHD43415.1	2	c.19110C>T	p.Phe6370Phe	synonymous_variant	LOW	0.997494	2903.41	3347	Patient2_210122
B.1.1.529 (BA.1.17)	21762	CTATACATG	TTA	MIXED	Homozygous	S	QHD43416.1	1	c.200_208delCTATACATGinsTTA	p.Ala67_Val70delinsValIle	missense_variant&disruptive_inframe_deletion	MODERATE	1.0	2945.09	3538	Patient2_210122
B.1.1.529 (BA.1.17	21986	GGTGTTTATT	G	DEL	Homozygous	S	QHD43416.1	1	c.425_433delGTGTTTATT	p.Gly142_Tyr145delinsAsp	disruptive_inframe_deletion	MODERATE	1.0	527.588	70	Patient2_210122

)															
B.1.1.529 (BA.1.17)	22193	AATT	A	DEL	Homozygous	S	QHD43416.1	1	c.632_634delATT	p.Asn211_Leu212delinsIle	disruptive_inframe_deletion	MODERATE	1.0	2968.15	3399	Patient 2_210122
B.1.1.529 (BA.1.17)	22204	T	TGAGCCAGAA	INS	Homozygous	S	QHD43416.1	1	c.644_645insGCCAGAAGA	p.Arg214_Asp215insGluProGlu	disruptive_inframe_insertion	MODERATE	0.979899	2745.59	3409	Patient 2_210122
B.1.1.529 (BA.1.17)	22578	G	A	SNP	Homozygous	S	QHD43416.1	1	c.1016G>A	p.Gly339Asp	missense_variant	MODERATE	0.997494	2941.48	4924	Patient 2_210122
B.1.1.529 (BA.1.17)	22673	TC	CT	MNP	Homozygous	S	QHD43416.1	1	c.1111_1112delTCinsCT	p.Ser371Leu	missense_variant	MODERATE	0.992443	2846.85	2806	Patient 2_210122
B.1.1.529 (BA.1.17)	22679	T	C	SNP	Homozygous	S	QHD43416.1	1	c.1117T>C	p.Ser373Pro	missense_variant	MODERATE	0.9975	2948.15	2815	Patient 2_210122
B.1.1.529 (BA.1.17)	22686	C	T	SNP	Homozygous	S	QHD43416.1	1	c.1124C>T	p.Ser375Phe	missense_variant	MODERATE	0.997494	2908.37	1667	Patient 2_210122
B.1.1.529 (BA.1.17)	22813	G	T	SNP	Homozygous	S	QHD43416.1	1	c.1251G>T	p.Lys417Asn	missense_variant	MODERATE	1.0	2982.14	6858	Patient 2_210122
B.1.1.529 (BA.1.17)	22882	T	G	SNP	Homozygous	S	QHD43416.1	1	c.1320T>G	p.Asn440Lys	missense_variant	MODERATE	1.0	2982.2	11191	Patient 2_210122
B.1.1.529 (BA.1.17)	22898	G	A	SNP	Homozygous	S	QHD43416.1	1	c.1336G>A	p.Gly446Ser	missense_variant	MODERATE	0.997494	2940.33	11232	Patient 2_210122
B.1.1.529 (BA.1.17)	22992	GCAC	ACAA	MNP	Homozygous	S	QHD43416.1	1	c.1430_1433delGCACinsACAA	p.SerThr477AsnLys	missense_variant	MODERATE	1.0	2969.73	6162	Patient 2_210122
B.1.1.529 (BA.1.17)	23013	A	C	SNP	Homozygous	S	QHD43416.1	1	c.1451A>C	p.Glu484Ala	missense_variant	MODERATE	1.0	2949.67	6140	Patient 2_210122
B.1.1.529 (BA.1.17)	23040	A	G	SNP	Homozygous	S	QHD43416.1	1	c.1478A>G	p.Gln493Arg	missense_variant	MODERATE	1.0	2842.59	6043	Patient 2_210122

B.1.1.529 (BA.1.17)	23202	C	A	SNP	Homozygous	S	QHD43416.1	1	c.1640C>A	p.Thr547Lys	missense_variant	MODERATE	1.0	2975.02	2046	Patient 2_210122
B.1.1.529 (BA.1.17)	23403	A	G	SNP	Homozygous	S	QHD43416.1	1	c.1841A>G	p.Asp614Gly	missense_variant	MODERATE	1.0	2981.97	8906	Patient 2_210122
B.1.1.529 (BA.1.17)	23422	C	T	SNP	Homozygous	S	QHD43416.1	1	c.1860C>T	p.Val620Val	synonymous_variant	LOW	0.997487	2936.38	8940	Patient 2_210122
B.1.1.529 (BA.1.17)	23525	C	T	SNP	Homozygous	S	QHD43416.1	1	c.1963C>T	p.His655Tyr	missense_variant	MODERATE	0.995	2919.61	9963	Patient 2_210122
B.1.1.529 (BA.1.17)	23599	T	G	SNP	Homozygous	S	QHD43416.1	1	c.2037T>G	p.Asn679Lys	missense_variant	MODERATE	1.0	2974.75	7837	Patient 2_210122
B.1.1.529 (BA.1.17)	23604	C	A	SNP	Homozygous	S	QHD43416.1	1	c.2042C>A	p.Pro681His	missense_variant	MODERATE	1.0	2974.76	7841	Patient 2_210122
B.1.1.529 (BA.1.17)	23854	C	A	SNP	Homozygous	S	QHD43416.1	1	c.2292C>A	p.Asn764Lys	missense_variant	MODERATE	1.0	2974.42	4308	Patient 2_210122
B.1.1.529 (BA.1.17)	23948	G	T	SNP	Homozygous	S	QHD43416.1	1	c.2386G>T	p.Asp796Tyr	missense_variant	MODERATE	1.0	2974.75	2591	Patient 2_210122
B.1.1.529 (BA.1.17)	24130	C	A	SNP	Homozygous	S	QHD43416.1	1	c.2568C>A	p.Asn856Lys	missense_variant	MODERATE	1.0	2961.13	4250	Patient 2_210122
B.1.1.529 (BA.1.17)	24424	A	T	SNP	Homozygous	S	QHD43416.1	1	c.2862A>T	p.Gln954His	missense_variant	MODERATE	0.997494	2373.79	3921	Patient 2_210122
B.1.1.529 (BA.1.17)	24469	T	A	SNP	Homozygous	S	QHD43416.1	1	c.2907T>A	p.Asn969Lys	missense_variant	MODERATE	0.994987	2910.7	10275	Patient 2_210122
B.1.1.529 (BA.1.17)	24503	C	T	SNP	Homozygous	S	QHD43416.1	1	c.2941C>T	p.Leu981Phe	missense_variant	MODERATE	0.9875	2277.74	10469	Patient 2_210122
B.1.1.529 (BA.1.17	25000	C	T	SNP	Homozygous	S	QHD43416.1	1	c.3438C>T	p.Asp1146Asp	synonymous_variant	LOW	1.0	2951.59	7061	Patient 2_210122

)																	
B.1.1.529 (BA.1.17)	25584	C	T	SNP	Homozygous	ORF3a	QHD43417.1	1	c.192C>T	p.Thr64Thr	synonymous_variant	LOW	0.9975	2948.9	5551	Patient 2_210122
B.1.1.529 (BA.1.17)	26270	C	T	SNP	Homozygous	E	QHD43418.1	1	c.26C>T	p.Thr9Ile	missense_variant	MODERATE	1.0	2982.15	14153	Patient 2_210122
B.1.1.529 (BA.1.17)	26530	A	G	SNP	Homozygous	M	QHD43419.1	1	c.8A>G	p.Asp3Gly	missense_variant	MODERATE	1.0	2982.21	3554	Patient 2_210122
B.1.1.529 (BA.1.17)	26577	C	G	SNP	Homozygous	M	QHD43419.1	1	c.55C>G	p.Gln19Glu	missense_variant	MODERATE	0.997494	2932.22	1603	Patient 2_210122
B.1.1.529 (BA.1.17)	26709	G	A	SNP	Homozygous	M	QHD43419.1	1	c.187G>A	p.Ala63Thr	missense_variant	MODERATE	0.9975	2948.9	4185	Patient 2_210122
B.1.1.529 (BA.1.17)	27259	A	C	SNP	Homozygous	ORF6	QHD43420.1	1	c.58A>C	p.Arg20Arg	synonymous_variant	LOW	1.0	2959.3	6686	Patient 2_210122
B.1.1.529 (BA.1.17)	27807	C	T	SNP	Homozygous	ORF8	QHD43422.1	-1	c.-87C>T	.	upstream_gene_variant	MODIFIER	1.0	2982.21	6873	Patient 2_210122
B.1.1.529 (BA.1.17)	28271	A	T	SNP	Homozygous	N	QHD43423.2	-1	c.-3A>T	.	upstream_gene_variant	MODIFIER	0.9825	2795.94	15309	Patient 2_210122
B.1.1.529 (BA.1.17)	28311	C	T	SNP	Homozygous	N	QHD43423.2	1	c.38C>T	p.Pro13Leu	missense_variant	MODERATE	0.995	2352.54	15320	Patient 2_210122
B.1.1.529 (BA.1.17)	28361	GGAGAACGCA	G	DEL	Homozygous	N	QHD43423.2	1	c.90_98delAGAACGCAG	p.Glu31_Ser33del	disruptive_inframe_deletion	MODERATE	1.0	2922.71	4701	Patient 2_210122
B.1.1.529 (BA.1.17)	28881	GGG	AAC	MNP	Homozygous	N	QHD43423.2	1	c.608_610delGGGinsAAC	p.ArgGly203LysArg	missense_variant	MODERATE	1.0	2934.8	3726	Patient 2_210122
B.1.1.529 (BA.1.17)	241	C	T	SNP	Homozygous	orf1ab	QHD43415.1	-1	c.-25C>T	.	upstream_gene_variant	MODIFIER	1.0	2968.2	2082	Patient 2_120222

B.1.1.529 (BA.1.17)	2832	A	G	SNP	Homozygous	orf1ab	QHD43415.1	1	c.2567A>G	p.Lys856Arg	missense_variant	MODERATE	1.0	2974.58	5146	Patient 2_120222
B.1.1.529 (BA.1.17)	3037	C	T	SNP	Homozygous	orf1ab	QHD43415.1	1	c.2772C>T	p.Phe924Phe	synonymous_variant	LOW	0.9975	2921.48	4934	Patient 2_120222
B.1.1.529 (BA.1.17)	5386	T	G	SNP	Homozygous	orf1ab	QHD43415.1	1	c.5121T>G	p.Ala1707Ala	synonymous_variant	LOW	1.0	2974.76	3829	Patient 2_120222
B.1.1.529 (BA.1.17)	5672	C	T	SNP	Homozygous	orf1ab	QHD43415.1	1	c.5407C>T	p.Pro1803Ser	missense_variant	MODERATE	0.987469	2833.04	3851	Patient 2_120222
B.1.1.529 (BA.1.17)	5924	G	A	SNP	Homozygous	orf1ab	QHD43415.1	1	c.5659G>A	p.Val1887Ile	missense_variant	MODERATE	0.9975	2933.23	3515	Patient 2_120222
B.1.1.529 (BA.1.17)	6512	AGTT	A	DEL	Homozygous	orf1ab	QHD43415.1	1	c.6248_6250delGTT	p.Ser2083_Leu2084delinsIle	disruptive_inframe_deletion	MODERATE	1.0	2928.82	1980	Patient 2_120222
B.1.1.529 (BA.1.17)	8393	G	A	SNP	Homozygous	orf1ab	QHD43415.1	1	c.8128G>A	p.Ala2710Thr	missense_variant	MODERATE	1.0	2982.16	3835	Patient 2_120222
B.1.1.529 (BA.1.17)	10029	C	T	SNP	Homozygous	orf1ab	QHD43415.1	1	c.9764C>T	p.Thr3255Ile	missense_variant	MODERATE	0.997487	2922.63	3766	Patient 2_120222
B.1.1.529 (BA.1.17)	10449	C	A	SNP	Homozygous	orf1ab	QHD43415.1	1	c.10184C>A	p.Pro3395His	missense_variant	MODERATE	0.997436	2875.65	2078	Patient 2_120222
B.1.1.529 (BA.1.17)	11282	AGTTTGTCTG	A	DEL	Homozygous	orf1ab	QHD43415.1	1	c.11022_11030delGTCTGGTTT	p.Leu3674_Gly3676del	disruptive_inframe_deletion	MODERATE	1.0	2917.49	3599	Patient 2_120222
B.1.1.529 (BA.1.17)	11537	A	G	SNP	Homozygous	orf1ab	QHD43415.1	1	c.11272A>G	p.Ile3758Val	missense_variant	MODERATE	1.0	2981.64	3784	Patient 2_120222
B.1.1.529 (BA.1.17)	13195	T	C	SNP	Homozygous	orf1ab	QHD43415.1	1	c.12930T>C	p.Val4310Val	synonymous_variant	LOW	1.0	2982.17	6090	Patient 2_120222
B.1.1.529 (BA.1.17	14408	C	T	SNP	Homozygous	orf1ab	QHD43415.1	2	c.14144C>T	p.Pro4715Leu	missense_variant	MODERATE	0.997494	2941.47	3242	Patient 2_120222

)															
B.1.1.529 (BA.1.17)	15240	C	T	SNP	Homozygous	orf1ab	QHD43415.1	2	c.14976C>T	p.Asn4992Asn	synonymous_variant	LOW	1.0	2980.31	6673	Patient 2_120222
B.1.1.529 (BA.1.17)	18163	A	G	SNP	Homozygous	orf1ab	QHD43415.1	2	c.17899A>G	p.Ile5967Val	missense_variant	MODERATE	1.0	2974.76	3093	Patient 2_120222
B.1.1.529 (BA.1.17)	19374	C	T	SNP	Homozygous	orf1ab	QHD43415.1	2	c.19110C>T	p.Phe6370Phe	synonymous_variant	LOW	1.0	2906.16	3145	Patient 2_120222
B.1.1.529 (BA.1.17)	21762	CTATACATG	TTA	MIXED	Homozygous	S	QHD43416.1	1	c.200_208delCTATACATGinsTTA	p.Ala67_Val70delinsValIle	missense_variant&disruptive_inframe_deletion	MODERATE	1.0	2913.03	2299	Patient 2_120222
B.1.1.529 (BA.1.17)	22193	AATT	A	DEL	Homozygous	S	QHD43416.1	1	c.632_634delATT	p.Asn211_Leu212delinsIle	disruptive_inframe_deletion	MODERATE	1.0	2974.73	2707	Patient 2_120222
B.1.1.529 (BA.1.17)	22204	T	TGAGCCAGAA	INS	Homozygous	S	QHD43416.1	1	c.644_645insGCCAGAAGA	p.Arg214_Asp215insGluProGlu	disruptive_inframe_insertion	MODERATE	0.989975	2851.81	2713	Patient 2_120222
B.1.1.529 (BA.1.17)	22578	G	A	SNP	Homozygous	S	QHD43416.1	1	c.1016G>A	p.Gly339Asp	missense_variant	MODERATE	1.0	2974.78	1210	Patient 2_120222
B.1.1.529 (BA.1.17)	22581	A	C	SNP	Homozygous	S	QHD43416.1	1	c.1019A>C	p.Glu340Ala	missense_variant	MODERATE	0.909774	1719.65	251	Patient 2_120222
B.1.1.529 (BA.1.17)	22582	A	C	SNP	Heterozygous	S	QHD43416.1	1	c.1020A>C	p.Glu340Asp	missense_variant	MODERATE	0.410359	791.641	251	Patient 2_120222
B.1.1.529 (BA.1.17)	22673	TC	CT	MNP	Homozygous	S	QHD43416.1	1	c.1111_1112delTCinsCT	p.Ser371Leu	missense_variant	MODERATE	0.994975	2905.1	1413	Patient 2_120222
B.1.1.529 (BA.1.17)	22679	T	C	SNP	Homozygous	S	QHD43416.1	1	c.1117T>C	p.Ser373Pro	missense_variant	MODERATE	1.0	2980.21	1416	Patient 2_120222
B.1.1.529 (BA.1.17)	22686	C	T	SNP	Homozygous	S	QHD43416.1	1	c.1124C>T	p.Ser375Phe	missense_variant	MODERATE	0.992481	2847.84	1117	Patient 2_120222

B.1.1.529 (BA.1.17)	22813	G	T	SNP	Homozygous	S	QHD43416.1	1	c.1251G>T	p.Lys417Asn	missense_variant	MODERATE	1.0	2982.17	4197	Patient 2_120222
B.1.1.529 (BA.1.17)	22882	T	G	SNP	Homozygous	S	QHD43416.1	1	c.1320T>G	p.Asn440Lys	missense_variant	MODERATE	1.0	2974.77	5223	Patient 2_120222
B.1.1.529 (BA.1.17)	22898	G	A	SNP	Homozygous	S	QHD43416.1	1	c.1336G>A	p.Gly446Ser	missense_variant	MODERATE	0.9975	2948.75	5208	Patient 2_120222
B.1.1.529 (BA.1.17)	22992	GCAC	ACAA	MNP	Homozygous	S	QHD43416.1	1	c.1430_1433delGCACinsACAA	p.SerThr477AsnLys	missense_variant	MODERATE	1.0	2928.64	2214	Patient 2_120222
B.1.1.529 (BA.1.17)	23013	A	C	SNP	Homozygous	S	QHD43416.1	1	c.1451A>C	p.Glu484Ala	missense_variant	MODERATE	1.0	2972.43	2208	Patient 2_120222
B.1.1.529 (BA.1.17)	23040	A	G	SNP	Homozygous	S	QHD43416.1	1	c.1478A>G	p.Gln493Arg	missense_variant	MODERATE	0.971649	2612.37	2134	Patient 2_120222
B.1.1.529 (BA.1.17)	23202	C	A	SNP	Homozygous	S	QHD43416.1	1	c.1640C>A	p.Thr547Lys	missense_variant	MODERATE	1.0	2966.75	2105	Patient 2_120222
B.1.1.529 (BA.1.17)	23403	A	G	SNP	Homozygous	S	QHD43416.1	1	c.1841A>G	p.Asp614Gly	missense_variant	MODERATE	1.0	2972.27	5996	Patient 2_120222
B.1.1.529 (BA.1.17)	23422	C	T	SNP	Homozygous	S	QHD43416.1	1	c.1860C>T	p.Val620Val	synonymous_variant	LOW	0.9975	2948.09	6041	Patient 2_120222
B.1.1.529 (BA.1.17)	23525	C	T	SNP	Homozygous	S	QHD43416.1	1	c.1963C>T	p.His655Tyr	missense_variant	MODERATE	1.0	2952.98	7904	Patient 2_120222
B.1.1.529 (BA.1.17)	23599	T	G	SNP	Homozygous	S	QHD43416.1	1	c.2037T>G	p.Asn679Lys	missense_variant	MODERATE	1.0	2974.77	6581	Patient 2_120222
B.1.1.529 (BA.1.17)	23604	C	A	SNP	Homozygous	S	QHD43416.1	1	c.2042C>A	p.Pro681His	missense_variant	MODERATE	0.994987	2346.78	6583	Patient 2_120222
B.1.1.529 (BA.1.17	23854	C	A	SNP	Homozygous	S	QHD43416.1	1	c.2292C>A	p.Asn764Lys	missense_variant	MODERATE	0.997487	2909.03	1691	Patient 2_120222

)															
B.1.1.529 (BA.1.17)	23948	G	T	SNP	Homozygous	S	QHD43416.1	1	c.2386G>T	p.Asp796Tyr	missense_variant	MODERATE	1.0	2982.17	1366	Patient 2_120222
B.1.1.529 (BA.1.17)	24130	C	A	SNP	Homozygous	S	QHD43416.1	1	c.2568C>A	p.Asn856Lys	missense_variant	MODERATE	1.0	2942.62	2170	Patient 2_120222
B.1.1.529 (BA.1.17)	24424	A	T	SNP	Homozygous	S	QHD43416.1	1	c.2862A>T	p.Gln954His	missense_variant	MODERATE	0.992481	2320.36	1815	Patient 2_120222
B.1.1.529 (BA.1.17)	24469	T	A	SNP	Homozygous	S	QHD43416.1	1	c.2907T>A	p.Asn969Lys	missense_variant	MODERATE	0.987469	2792.0	5563	Patient 2_120222
B.1.1.529 (BA.1.17)	24503	C	T	SNP	Homozygous	S	QHD43416.1	1	c.2941C>T	p.Leu981Phe	missense_variant	MODERATE	0.969925	2670.04	5741	Patient 2_120222
B.1.1.529 (BA.1.17)	25000	C	T	SNP	Homozygous	S	QHD43416.1	1	c.3438C>T	p.Asp1146Asp	synonymous_variant	LOW	0.9975	2949.52	5337	Patient 2_120222
B.1.1.529 (BA.1.17)	25584	C	T	SNP	Homozygous	ORF3a	QHD43417.1	1	c.192C>T	p.Thr64Thr	synonymous_variant	LOW	0.994975	2340.04	3717	Patient 2_120222
B.1.1.529 (BA.1.17)	26270	C	T	SNP	Homozygous	E	QHD43418.1	1	c.26C>T	p.Thr9Ile	missense_variant	MODERATE	1.0	2982.2	8205	Patient 2_120222
B.1.1.529 (BA.1.17)	26530	A	G	SNP	Homozygous	M	QHD43419.1	1	c.8A>G	p.Asp3Gly	missense_variant	MODERATE	1.0	2974.28	5045	Patient 2_120222
B.1.1.529 (BA.1.17)	26577	C	G	SNP	Homozygous	M	QHD43419.1	1	c.55C>G	p.Gln19Glu	missense_variant	MODERATE	1.0	2982.17	2713	Patient 2_120222
B.1.1.529 (BA.1.17)	26709	G	A	SNP	Homozygous	M	QHD43419.1	1	c.187G>A	p.Ala63Thr	missense_variant	MODERATE	1.0	2982.21	2992	Patient 2_120222
B.1.1.529 (BA.1.17)	27259	A	C	SNP	Homozygous	ORF6	QHD43420.1	1	c.58A>C	p.Arg20Arg	synonymous_variant	LOW	0.997487	2935.3	4148	Patient 2_120222

B.1.1.529 (BA.1.17)	27807	C	T	SNP	Homozygous	ORF8	QHD43422.1	-1	c.-87C>T	.	upstream_gene_variant	MODIFIER	1.0	2982.21	7088	Patient2_120222
B.1.1.529 (BA.1.17)	28271	A	T	SNP	Homozygous	N	QHD43423.2	-1	c.-3A>T	.	upstream_gene_variant	MODIFIER	0.985	2798.84	7104	Patient2_120222
B.1.1.529 (BA.1.17)	28311	C	T	SNP	Homozygous	N	QHD43423.2	1	c.38C>T	p.Pro13Leu	missense_variant	MODERATE	0.9775	2744.79	7093	Patient2_120222
B.1.1.529 (BA.1.17)	28361	GGAGAACGCA	G	DEL	Homozygous	N	QHD43423.2	1	c.90_98delAGAACGCAG	p.Glu31_Ser33del	disruptive_inframe_deletion	MODERATE	1.0	2924.98	3555	Patient2_120222
B.1.1.529 (BA.1.17)	28881	GGG	AAC	MNP	Homozygous	N	QHD43423.2	1	c.608_610delGGGinsAAC	p.ArgGly203LysArg	missense_variant	MODERATE	1.0	2958.37	2792	Patient2_120222
B.1.1.529 (BA.2)	44	C	T	SNP	Homozygous	orf1ab	QHD43415.1	-1	c.-222C>T	.	upstream_gene_variant	MODIFIER	1.0	2970.91	7788	Patient4_060522
B.1.1.529 (BA.2)	241	C	T	SNP	Homozygous	orf1ab	QHD43415.1	-1	c.-25C>T	.	upstream_gene_variant	MODIFIER	0.997494	2932.6	527	Patient4_060522
B.1.1.529 (BA.2)	670	T	G	SNP	Homozygous	orf1ab	QHD43415.1	1	c.405T>G	p.Ser135Arg	missense_variant	MODERATE	0.995	2919.58	6649	Patient4_060522
B.1.1.529 (BA.2)	1738	G	T	SNP	Heterozygous	orf1ab	QHD43415.1	1	c.1473G>T	p.Val491Val	synonymous_variant	LOW	0.2675	51.813	8490	Patient4_060522
B.1.1.529 (BA.2)	2790	C	T	SNP	Homozygous	orf1ab	QHD43415.1	1	c.2525C>T	p.Thr842Ile	missense_variant	MODERATE	1.0	2915.82	4454	Patient4_060522
B.1.1.529 (BA.2)	3037	C	T	SNP	Homozygous	orf1ab	QHD43415.1	1	c.2772C>T	p.Phe924Phe	synonymous_variant	LOW	1.0	2944.56	6099	Patient4_060522
B.1.1.529 (BA.2)	4184	G	A	SNP	Homozygous	orf1ab	QHD43415.1	1	c.3919G>A	p.Gly1307Ser	missense_variant	MODERATE	0.9975	2948.9	4218	Patient4_060522
B.1.1.529 (BA.2)	4321	C	T	SNP	Homozygous	orf1ab	QHD43415.1	1	c.4056C>T	p.Ala1352Ala	synonymous_variant	LOW	0.9875	2830.53	6401	Patient4_060522
B.1.1.529 (BA.2)	4543	C	T	SNP	Homozygous	orf1ab	QHD43415.1	1	c.4278C>T	p.Thr1426Thr	synonymous_variant	LOW	0.9975	2948.9	9129	Patient4_060522
B.1.1.529 (BA.2)	7303	C	T	SNP	Homozygous	orf1ab	QHD43415.1	1	c.7038C>T	p.Ile2346Ile	synonymous_variant	LOW	1.0	2982.2	3304	Patient4_060522

B.1.1.529 (BA.2)	9344	C	T	SNP	Homozygous	orf1ab	QHD43415.1	1	c.9079C>T	p.Leu3027Phe	missense_variant	MODERATE	1.0	2981.48	5855	Patient4_060522
B.1.1.529 (BA.2)	9424	A	G	SNP	Homozygous	orf1ab	QHD43415.1	1	c.9159A>G	p.Val3053Val	synonymous_variant	LOW	1.0	2972.7	3171	Patient4_060522
B.1.1.529 (BA.2)	9534	C	T	SNP	Homozygous	orf1ab	QHD43415.1	1	c.9269C>T	p.Thr3090Ile	missense_variant	MODERATE	1.0	1227.96	165	Patient4_060522
B.1.1.529 (BA.2)	9866	C	T	SNP	Homozygous	orf1ab	QHD43415.1	1	c.9601C>T	p.Leu3201Phe	missense_variant	MODERATE	1.0	2971.27	8500	Patient4_060522
B.1.1.529 (BA.2)	10029	C	T	SNP	Homozygous	orf1ab	QHD43415.1	1	c.9764C>T	p.Thr3255Ile	missense_variant	MODERATE	0.992481	2885.25	7898	Patient4_060522
B.1.1.529 (BA.2)	10198	C	T	SNP	Homozygous	orf1ab	QHD43415.1	1	c.9933C>T	p.Asp3311Asp	synonymous_variant	LOW	1.0	2982.15	9217	Patient4_060522
B.1.1.529 (BA.2)	10447	GCC	ACA	MNP	Homozygous	orf1ab	QHD43415.1	1	c.10182_10184delGCCinsACA	p.Pro3395His	missense_variant	MODERATE	1.0	2968.72	3532	Patient4_060522
B.1.1.529 (BA.2)	11287	GTCTGGTTTT	G	DEL	Homozygous	orf1ab	QHD43415.1	1	c.11023_11031delTCTGGTTTT	p.Ser3675_Phe3677del	conservative_inframe_deletion	MODERATE	1.0	2947.64	9927	Patient4_060522
B.1.1.529 (BA.2)	12880	C	T	SNP	Homozygous	orf1ab	QHD43415.1	1	c.12615C>T	p.Ile4205Ile	synonymous_variant	LOW	0.9975	2948.64	4543	Patient4_060522
B.1.1.529 (BA.2)	14408	C	T	SNP	Homozygous	orf1ab	QHD43415.1	2	c.14144C>T	p.Pro4715Leu	missense_variant	MODERATE	0.9975	2947.3	5097	Patient4_060522
B.1.1.529 (BA.2)	15714	C	T	SNP	Homozygous	orf1ab	QHD43415.1	2	c.15450C>T	p.Leu5150Leu	synonymous_variant	LOW	1.0	2982.21	8759	Patient4_060522
B.1.1.529 (BA.2)	17410	C	T	SNP	Homozygous	orf1ab	QHD43415.1	2	c.17146C>T	p.Arg5716Cys	missense_variant	MODERATE	1.0	2974.76	6919	Patient4_060522
B.1.1.529 (BA.2)	18163	A	G	SNP	Homozygous	orf1ab	QHD43415.1	2	c.17899A>G	p.Ile5967Val	missense_variant	MODERATE	1.0	2982.09	2074	Patient4_060522
B.1.1.529 (BA.2)	18888	C	T	SNP	Homozygous	orf1ab	QHD43415.1	2	c.18624C>T	p.His6208His	synonymous_variant	LOW	0.9975	2948.89	5756	Patient4_060522
B.1.1.529 (BA.2)	19955	C	T	SNP	Homozygous	orf1ab	QHD43415.1	2	c.19691C>T	p.Thr6564Ile	missense_variant	MODERATE	1.0	2974.57	6765	Patient4_060522
B.1.1.529 (BA.2)	20055	A	G	SNP	Homozygous	orf1ab	QHD43415.1	2	c.19791A>G	p.Glu6597Glu	synonymous_variant	LOW	0.9975	2379.74	6176	Patient4_060522
B.1.1.529 (BA.2)	2091	G	A	SNP	Homozygou	orf1ab	QHD43415.1	2	c.20655G>A	p.Gln6885Gln	synonymous_variant	LOW	0.995	2919.6	7939	Patient4_0605

	9				s									2		22
B.1.1.529 (BA.2)	21618	C	T	SNP	Homozygous	S	QHD43416.1	1	c.56C>T	p.Thr19Ile	missense_variant	MODERATE	0.9975	2949.1	9208	Patient4_060522
B.1.1.529 (BA.2)	21632	TTACCCCCTG	T	DEL	Homozygous	S	QHD43416.1	1	c.71_79delTACCCCCTG	p.Leu24_Ala27delinsSer	disruptive_inframe_deletion	MODERATE	1.0	2937.58	9058	Patient4_060522
B.1.1.529 (BA.2)	21987	G	A	SNP	Homozygous	S	QHD43416.1	1	c.425G>A	p.Gly142Asp	missense_variant	MODERATE	1.0	2954.86	7226	Patient4_060522
B.1.1.529 (BA.2)	22200	T	G	SNP	Homozygous	S	QHD43416.1	1	c.638T>G	p.Val213Gly	missense_variant	MODERATE	1.0	2981.17	5857	Patient4_060522
B.1.1.529 (BA.2)	22578	G	A	SNP	Homozygous	S	QHD43416.1	1	c.1016G>A	p.Gly339Asp	missense_variant	MODERATE	1.0	2974.77	5705	Patient4_060522
B.1.1.529 (BA.2)	22674	C	T	SNP	Homozygous	S	QHD43416.1	1	c.1112C>T	p.Ser371Phe	missense_variant	MODERATE	0.994987	2339.82	2332	Patient4_060522
B.1.1.529 (BA.2)	22679	T	C	SNP	Homozygous	S	QHD43416.1	1	c.1117T>C	p.Ser373Pro	missense_variant	MODERATE	1.0	2981.89	2338	Patient4_060522
B.1.1.529 (BA.2)	22686	CCA	TCG	MNP	Homozygous	S	QHD43416.1	1	c.1124_1126delCCAinsTCG	p.SerThr375PheAla	missense_variant	MODERATE	1.0	2930.49	2253	Patient4_060522
B.1.1.529 (BA.2)	22775	G	A	SNP	Homozygous	S	QHD43416.1	1	c.1213G>A	p.Asp405Asn	missense_variant	MODERATE	1.0	2982.21	2296	Patient4_060522
B.1.1.529 (BA.2)	22786	A	C	SNP	Homozygous	S	QHD43416.1	1	c.1224A>C	p.Arg408Ser	missense_variant	MODERATE	1.0	2982.21	2291	Patient4_060522
B.1.1.529 (BA.2)	22813	G	T	SNP	Homozygous	S	QHD43416.1	1	c.1251G>T	p.Lys417Asn	missense_variant	MODERATE	1.0	2982.18	7954	Patient4_060522
B.1.1.529 (BA.2)	22882	T	G	SNP	Homozygous	S	QHD43416.1	1	c.1320T>G	p.Asn440Lys	missense_variant	MODERATE	1.0	2974.76	11128	Patient4_060522
B.1.1.529 (BA.2)	22992	GCAC	ACAA	MNP	Homozygous	S	QHD43416.1	1	c.1430_1433delGCACinsACAA	p.SerThr477AsnLys	missense_variant	MODERATE	1.0	2957.86	5408	Patient4_060522
B.1.1.529 (BA.2)	23013	A	C	SNP	Homozygous	S	QHD43416.1	1	c.1451A>C	p.Glu484Ala	missense_variant	MODERATE	1.0	2982.19	5399	Patient4_060522
B.1.1.529 (BA.2)	23040	A	G	SNP	Homozygous	S	QHD43416.1	1	c.1478A>G	p.Gln493Arg	missense_variant	MODERATE	0.994366	2580.16	5309	Patient4_060522
B.1.1.529 (BA.2)	23403	A	G	SNP	Homozygous	S	QHD43416.1	1	c.1841A>G	p.Asp614Gly	missense_variant	MODERATE	1.0	2981.13	8124	Patient4_060522

B.1.1.529 (BA.2)	23525	C	T	SNP	Homozygous	S	QHD43416.1	1	c.1963C>T	p.His655Tyr	missense_variant	MODERATE	1.0	2981.98	9844	Patient 4_060522
B.1.1.529 (BA.2)	23599	T	G	SNP	Homozygous	S	QHD43416.1	1	c.2037T>G	p.Asn679Lys	missense_variant	MODERATE	1.0	2982.21	8861	Patient 4_060522
B.1.1.529 (BA.2)	23604	C	A	SNP	Homozygous	S	QHD43416.1	1	c.2042C>A	p.Pro681His	missense_variant	MODERATE	1.0	2975.36	8863	Patient 4_060522
B.1.1.529 (BA.2)	23854	C	A	SNP	Homozygous	S	QHD43416.1	1	c.2292C>A	p.Asn764Lys	missense_variant	MODERATE	1.0	2959.69	2752	Patient 4_060522
B.1.1.529 (BA.2)	23948	G	T	SNP	Homozygous	S	QHD43416.1	1	c.2386G>T	p.Asp796Tyr	missense_variant	MODERATE	1.0	2974.75	2768	Patient 4_060522
B.1.1.529 (BA.2)	24424	A	T	SNP	Homozygous	S	QHD43416.1	1	c.2862A>T	p.Gln954His	missense_variant	MODERATE	1.0	2981.54	2309	Patient 4_060522
B.1.1.529 (BA.2)	24469	T	A	SNP	Homozygous	S	QHD43416.1	1	c.2907T>A	p.Asn969Lys	missense_variant	MODERATE	1.0	2930.64	6945	Patient 4_060522
B.1.1.529 (BA.2)	25000	C	T	SNP	Homozygous	S	QHD43416.1	1	c.3438C>T	p.Asp1146Asp	synonymous_variant	LOW	1.0	2979.74	7151	Patient 4_060522
B.1.1.529 (BA.2)	25584	C	T	SNP	Homozygous	ORF3a	QHD43417.1	1	c.192C>T	p.Thr64Thr	synonymous_variant	LOW	1.0	2965.35	5885	Patient 4_060522
B.1.1.529 (BA.2)	25587	C	T	SNP	Homozygous	ORF3a	QHD43417.1	1	c.195C>T	p.Leu65Leu	synonymous_variant	LOW	1.0	2982.12	5886	Patient 4_060522
B.1.1.529 (BA.2)	26060	C	T	SNP	Homozygous	ORF3a	QHD43417.1	1	c.668C>T	p.Thr223Ile	missense_variant	MODERATE	1.0	2960.41	5324	Patient 4_060522
B.1.1.529 (BA.2)	26270	C	T	SNP	Homozygous	E	QHD43418.1	1	c.26C>T	p.Thr9Ile	missense_variant	MODERATE	1.0	2982.21	12218	Patient 4_060522
B.1.1.529 (BA.2)	26577	C	G	SNP	Homozygous	M	QHD43419.1	1	c.55C>G	p.Gln19Glu	missense_variant	MODERATE	1.0	2974.75	3837	Patient 4_060522
B.1.1.529 (BA.2)	26709	G	A	SNP	Homozygous	M	QHD43419.1	1	c.187G>A	p.Ala63Thr	missense_variant	MODERATE	1.0	2982.21	4564	Patient 4_060522
B.1.1.529 (BA.2)	26858	C	T	SNP	Homozygous	M	QHD43419.1	1	c.336C>T	p.Phe112Phe	synonymous_variant	LOW	1.0	2982.11	4477	Patient 4_060522
B.1.1.529 (BA.2)	27002	C	T	SNP	Homozygous	M	QHD43419.1	1	c.480C>T	p.Asp160Asp	synonymous_variant	LOW	1.0	2982.21	7459	Patient 4_060522
B.1.1.529 (BA.2)	2725	A	C	SNP	Homozygou	ORF6	QHD43420.1	1	c.58A>C	p.Arg20Arg	synonymous_variant	LOW	1.0	2974.5	6857	Patient 4_0605

	9				s									7		22
B.1.1.529 (BA.2)	27382	GAT	CTC	MNP	Homozygous	ORF6	QHD43420.1	1	c.181_183delGATinsCTC	p.Asp61Leu	missense_variant	MODERATE	1.0	2935.96	3649	Patient 4_060522
B.1.1.529 (BA.2)	27494	C	T	SNP	Heterozygous	ORF7a	QHD43421.1	1	c.101C>T	p.Pro34Leu	missense_variant	MODERATE	0.407609	125.805	184	Patient 4_060522
B.1.1.529 (BA.2)	27807	C	T	SNP	Homozygous	ORF8	QHD43422.1	-1	c.-87C>T	.	upstream_gene_variant	MODIFIER	1.0	2982.21	9481	Patient 4_060522
B.1.1.529 (BA.2)	28271	A	T	SNP	Homozygous	N	QHD43423.2	-1	c.-3A>T	.	upstream_gene_variant	MODIFIER	1.0	2915.46	10170	Patient 4_060522
B.1.1.529 (BA.2)	28311	C	T	SNP	Homozygous	N	QHD43423.2	1	c.38C>T	p.Pro13Leu	missense_variant	MODERATE	0.99	2865.31	10175	Patient 4_060522
B.1.1.529 (BA.2)	28361	GGAGAACGCA	G	DEL	Homozygous	N	QHD43423.2	1	c.90_98delAGAACGCAG	p.Glu31_Ser33del	disruptive_inframe_deletion	MODERATE	1.0	2955.99	3895	Patient 4_060522
B.1.1.529 (BA.2)	28881	GGG	AAC	MNP	Homozygous	N	QHD43423.2	1	c.608_610delGGGinsAAC	p.ArgGly203LysArg	missense_variant	MODERATE	1.0	2967.34	1975	Patient 4_060522
B.1.1.529 (BA.2)	29250	C	T	SNP	Heterozygous	N	QHD43423.2	1	c.977C>T	p.Pro326Leu	missense_variant	MODERATE	0.7675	1438.82	2675	Patient 4_060522
B.1.1.529 (BA.2)	29510	A	C	SNP	Homozygous	N	QHD43423.2	1	c.1237A>C	p.Ser413Arg	missense_variant	MODERATE	1.0	2975.76	972	Patient 4_060522
B.1.1.529 (BA.2)	29733	CGAGGCCACGCGGAGTACGATCGAGTG	C	DEL	Homozygous	S	QHD43416.1	-1	c.*4350_*4375delGAGGCCACGCGGAGTACGATCGAGTG	.	downstream_gene_variant	MODIFIER	1.0	2952.87	1244	Patient 4_060522
B.1.1.529 (BA.2.9)	44	C	T	SNP	Homozygous	orf1ab	QHD43415.1	-1	c.-222C>T	.	upstream_gene_variant	MODIFIER	1.0	2974.95	7074	Patient 3_280322
B.1.1.529 (BA.2.9)	241	C	T	SNP	Homozygous	orf1ab	QHD43415.1	-1	c.-25C>T	.	upstream_gene_variant	MODIFIER	1.0	2967.05	406	Patient 3_280322
B.1.1.529 (BA.2.9)	670	T	G	SNP	Homozygous	orf1ab	QHD43415.1	1	c.405T>G	p.Ser135Arg	missense_variant	MODERATE	0.995	2919.63	7667	Patient 3_280322
B.1.1.529 (BA.2.9)	2790	C	T	SNP	Homozygous	orf1ab	QHD43415.1	1	c.2525C>T	p.Thr842Ile	missense_variant	MODERATE	1.0	2886.3	3822	Patient 3_280322
B.1.1.529 (BA.2.9)	3037	C	T	SNP	Homozygous	orf1ab	QHD43415.1	1	c.2772C>T	p.Phe924Phe	synonymous_variant	LOW	1.0	2964.64	6043	Patient 3_280322
B.1.1.529 (BA.2.9)	4184	G	A	SNP	Homozygous	orf1ab	QHD43415.1	1	c.3919G>A	p.Gly1307Ser	missense_variant	MODERATE	1.0	2982.17	5693	Patient 3_280322

B.1.1.529 (BA.2.9)	4321	C	T	SNP	Homozygous	orf1ab	QHD43415.1	1	c.4056C>T	p.Ala1352Ala	synonymous_variant	LOW	0.985	2814.89	6225	Patient 3_280322
B.1.1.529 (BA.2.9)	7101	C	T	SNP	Homozygous	orf1ab	QHD43415.1	1	c.6836C>T	p.Ala2279Val	missense_variant	MODERATE	1.0	2982.21	2035	Patient 3_280322
B.1.1.529 (BA.2.9)	9344	C	T	SNP	Homozygous	orf1ab	QHD43415.1	1	c.9079C>T	p.Leu3027Phe	missense_variant	MODERATE	1.0	2976.5	6054	Patient 3_280322
B.1.1.529 (BA.2.9)	9424	A	G	SNP	Homozygous	orf1ab	QHD43415.1	1	c.9159A>G	p.Val3053Val	synonymous_variant	LOW	1.0	2981.06	4095	Patient 3_280322
B.1.1.529 (BA.2.9)	9534	C	T	SNP	Homozygous	orf1ab	QHD43415.1	1	c.9269C>T	p.Thr3090Ile	missense_variant	MODERATE	1.0	1173.6	158	Patient 3_280322
B.1.1.529 (BA.2.9)	9866	C	T	SNP	Homozygous	orf1ab	QHD43415.1	1	c.9601C>T	p.Leu3201Phe	missense_variant	MODERATE	1.0	2969.34	6594	Patient 3_280322
B.1.1.529 (BA.2.9)	10029	C	T	SNP	Homozygous	orf1ab	QHD43415.1	1	c.9764C>T	p.Thr3255Ile	missense_variant	MODERATE	0.994987	2912.33	7027	Patient 3_280322
B.1.1.529 (BA.2.9)	10198	C	T	SNP	Homozygous	orf1ab	QHD43415.1	1	c.9933C>T	p.Asp3311Asp	synonymous_variant	LOW	0.997494	2941.3	7750	Patient 3_280322
B.1.1.529 (BA.2.9)	10447	GCC	ACA	MNP	Homozygous	orf1ab	QHD43415.1	1	c.10182_10184delGCCinsACA	p.Pro3395His	missense_variant	MODERATE	1.0	2967.27	3632	Patient 3_280322
B.1.1.529 (BA.2.9)	11287	GTCTGGTTTT	G	DEL	Homozygous	orf1ab	QHD43415.1	1	c.11023_11031delTCTGGTTTT	p.Ser3675_Phe3677del	conservative_inframe_deletion	MODERATE	1.0	2959.8	8241	Patient 3_280322
B.1.1.529 (BA.2.9)	12880	C	T	SNP	Homozygous	orf1ab	QHD43415.1	1	c.12615C>T	p.Ile4205Ile	synonymous_variant	LOW	1.0	2982.17	3826	Patient 3_280322
B.1.1.529 (BA.2.9)	14408	C	T	SNP	Homozygous	orf1ab	QHD43415.1	2	c.14144C>T	p.Pro4715Leu	missense_variant	MODERATE	0.992481	2884.38	2447	Patient 3_280322
B.1.1.529 (BA.2.9)	15714	C	T	SNP	Homozygous	orf1ab	QHD43415.1	2	c.15450C>T	p.Leu5150Leu	synonymous_variant	LOW	1.0	2982.21	5866	Patient 3_280322
B.1.1.529 (BA.2.9)	17410	C	T	SNP	Homozygous	orf1ab	QHD43415.1	2	c.17146C>T	p.Arg5716Cys	missense_variant	MODERATE	0.9975	2948.9	9123	Patient 3_280322
B.1.1.529 (BA.2.9)	18163	A	G	SNP	Homozygous	orf1ab	QHD43415.1	2	c.17899A>G	p.Ile5967Val	missense_variant	MODERATE	1.0	2982.19	1803	Patient 3_280322
B.1.1.529 (BA.2.9)	18636	G	T	SNP	Homozygous	orf1ab	QHD43415.1	2	c.18372G>T	p.Val6124Val	synonymous_variant	LOW	0.9975	2379.91	2550	Patient 3_280322
B.1.1.529	1947	T	A	SNP	Homozygou	orf1ab	QHD43415.1	2	c.19210T>A	p.Leu6404Ile	missense_variant	MODERATE	1.0	2948.4	4254	Patient 3_2803

(BA.2.9)	4				s									9		22
B.1.1.529 (BA.2.9)	19955	C	T	SNP	Homozygous	orf1ab	QHD43415.1	2	c.19691C>T	p.Thr6564Ile	missense_variant	MODERATE	1.0	2980.36	5064	Patient 3_280322
B.1.1.529 (BA.2.9)	20055	A	G	SNP	Homozygous	orf1ab	QHD43415.1	2	c.19791A>G	p.Glu6597Glu	synonymous_variant	LOW	1.0	2935.12	5195	Patient 3_280322
B.1.1.529 (BA.2.9)	21618	C	T	SNP	Homozygous	S	QHD43416.1	1	c.56C>T	p.Thr19Ile	missense_variant	MODERATE	1.0	2982.21	5905	Patient 3_280322
B.1.1.529 (BA.2.9)	21632	TTACCCCCTG	T	DEL	Homozygous	S	QHD43416.1	1	c.71_79delTACCCCCTG	p.Leu24_Ala27delinsSer	disruptive_inframe_deletion	MODERATE	1.0	2935.46	5825	Patient 3_280322
B.1.1.529 (BA.2.9)	21987	G	A	SNP	Homozygous	S	QHD43416.1	1	c.425G>A	p.Gly142Asp	missense_variant	MODERATE	0.9975	2948.87	4416	Patient 3_280322
B.1.1.529 (BA.2.9)	22200	T	G	SNP	Homozygous	S	QHD43416.1	1	c.638T>G	p.Val213Gly	missense_variant	MODERATE	1.0	2973.75	4240	Patient 3_280322
B.1.1.529 (BA.2.9)	22578	G	A	SNP	Homozygous	S	QHD43416.1	1	c.1016G>A	p.Gly339Asp	missense_variant	MODERATE	0.9975	2948.9	4524	Patient 3_280322
B.1.1.529 (BA.2.9)	22674	C	T	SNP	Homozygous	S	QHD43416.1	1	c.1112C>T	p.Ser371Phe	missense_variant	MODERATE	0.9975	2379.37	1854	Patient 3_280322
B.1.1.529 (BA.2.9)	22679	T	C	SNP	Homozygous	S	QHD43416.1	1	c.1117T>C	p.Ser373Pro	missense_variant	MODERATE	0.985	2813.33	1848	Patient 3_280322
B.1.1.529 (BA.2.9)	22686	CCA	TCG	MNP	Homozygous	S	QHD43416.1	1	c.1124_1126delCCAinsTCG	p.SerThr375PheAla	missense_variant	MODERATE	1.0	2961.48	1601	Patient 3_280322
B.1.1.529 (BA.2.9)	22775	G	A	SNP	Homozygous	S	QHD43416.1	1	c.1213G>A	p.Asp405Asn	missense_variant	MODERATE	0.9975	2943.38	1618	Patient 3_280322
B.1.1.529 (BA.2.9)	22786	A	C	SNP	Homozygous	S	QHD43416.1	1	c.1224A>C	p.Arg408Ser	missense_variant	MODERATE	1.0	2982.12	1617	Patient 3_280322
B.1.1.529 (BA.2.9)	22792	C	T	SNP	Homozygous	S	QHD43416.1	1	c.1230C>T	p.Ile410Ile	synonymous_variant	LOW	0.9975	2948.82	1610	Patient 3_280322
B.1.1.529 (BA.2.9)	22813	G	T	SNP	Homozygous	S	QHD43416.1	1	c.1251G>T	p.Lys417Asn	missense_variant	MODERATE	0.9975	2945.62	1690	Patient 3_280322
B.1.1.529 (BA.2.9)	22882	T	G	SNP	Homozygous	S	QHD43416.1	1	c.1320T>G	p.Asn440Lys	missense_variant	MODERATE	1.0	2982.2	7017	Patient 3_280322
B.1.1.529 (BA.2.9)	22992	GCAC	ACAA	MNP	Homozygous	S	QHD43416.1	1	c.1430_1433delGCACinsACAA	p.SerThr477AsnLys	missense_variant	MODERATE	1.0	2974.76	6976	Patient 3_280322

B.1.1.529 (BA.2.9)	23013	A	C	SNP	Homozygous	S	QHD43416.1	1	c.1451A>C	p.Glu484Ala	missense_variant	MODERATE	0.9975	2950.47	6959	Patient3_280322
B.1.1.529 (BA.2.9)	23040	A	G	SNP	Homozygous	S	QHD43416.1	1	c.1478A>G	p.Gln493Arg	missense_variant	MODERATE	0.997389	2819.27	6913	Patient3_280322
B.1.1.529 (BA.2.9)	23403	A	G	SNP	Homozygous	S	QHD43416.1	1	c.1841A>G	p.Asp614Gly	missense_variant	MODERATE	1.0	2977.34	8123	Patient3_280322
B.1.1.529 (BA.2.9)	23410	C	T	SNP	Homozygous	S	QHD43416.1	1	c.1848C>T	p.Asn616Asn	synonymous_variant	LOW	1.0	2982.2	8135	Patient3_280322
B.1.1.529 (BA.2.9)	23525	C	T	SNP	Homozygous	S	QHD43416.1	1	c.1963C>T	p.His655Tyr	missense_variant	MODERATE	1.0	2982.09	8971	Patient3_280322
B.1.1.529 (BA.2.9)	23599	T	G	SNP	Homozygous	S	QHD43416.1	1	c.2037T>G	p.Asn679Lys	missense_variant	MODERATE	0.9975	2948.89	7779	Patient3_280322
B.1.1.529 (BA.2.9)	23604	C	A	SNP	Homozygous	S	QHD43416.1	1	c.2042C>A	p.Pro681His	missense_variant	MODERATE	1.0	2981.7	7778	Patient3_280322
B.1.1.529 (BA.2.9)	23854	C	A	SNP	Homozygous	S	QHD43416.1	1	c.2292C>A	p.Asn764Lys	missense_variant	MODERATE	0.9975	2373.35	3080	Patient3_280322
B.1.1.529 (BA.2.9)	23948	G	T	SNP	Homozygous	S	QHD43416.1	1	c.2386G>T	p.Asp796Tyr	missense_variant	MODERATE	1.0	2982.01	2446	Patient3_280322
B.1.1.529 (BA.2.9)	24424	A	T	SNP	Homozygous	S	QHD43416.1	1	c.2862A>T	p.Gln954His	missense_variant	MODERATE	0.9975	2380.16	3492	Patient3_280322
B.1.1.529 (BA.2.9)	24469	T	A	SNP	Homozygous	S	QHD43416.1	1	c.2907T>A	p.Asn969Lys	missense_variant	MODERATE	0.9975	2886.7	8616	Patient3_280322
B.1.1.529 (BA.2.9)	25000	C	T	SNP	Homozygous	S	QHD43416.1	1	c.3438C>T	p.Asp1146Asp	synonymous_variant	LOW	0.995	2916.7	6039	Patient3_280322
B.1.1.529 (BA.2.9)	25584	C	T	SNP	Homozygous	ORF3a	QHD43417.1	1	c.192C>T	p.Thr64Thr	synonymous_variant	LOW	0.992481	2884.93	2772	Patient3_280322
B.1.1.529 (BA.2.9)	25624	C	T	SNP	Homozygous	ORF3a	QHD43417.1	1	c.232C>T	p.His78Tyr	missense_variant	MODERATE	0.997487	2924.32	4109	Patient3_280322
B.1.1.529 (BA.2.9)	26060	C	T	SNP	Homozygous	ORF3a	QHD43417.1	1	c.668C>T	p.Thr223Ile	missense_variant	MODERATE	1.0	2982.21	4567	Patient3_280322
B.1.1.529 (BA.2.9)	26270	C	T	SNP	Homozygous	E	QHD43418.1	1	c.26C>T	p.Thr9Ile	missense_variant	MODERATE	1.0	2970.76	12614	Patient3_280322
B.1.1.529	2657	C	G	SNP	Homozygou	M	QHD43419.1	1	c.55C>G	p.Gln19Glu	missense_variant	MODERATE	1.0	2982.2	2277	Patient3_2803

(BA.2.9)	7				s									1		22
B.1.1.529 (BA.2.9)	26709	G	A	SNP	Homozygous	M	QHD43419.1	1	c.187G>A	p.Ala63Thr	missense_variant	MODERATE	0.9975	2948.91	4464	Patient3_280322
B.1.1.529 (BA.2.9)	26753	C	T	SNP	Homozygous	M	QHD43419.1	1	c.231C>T	p.Thr77Thr	synonymous_variant	LOW	0.995	2919.66	4482	Patient3_280322
B.1.1.529 (BA.2.9)	26858	C	T	SNP	Homozygous	M	QHD43419.1	1	c.336C>T	p.Phe112Phe	synonymous_variant	LOW	0.9975	2948.83	4686	Patient3_280322
B.1.1.529 (BA.2.9)	27259	A	C	SNP	Homozygous	ORF6	QHD43420.1	1	c.58A>C	p.Arg20Arg	synonymous_variant	LOW	1.0	2982.08	6270	Patient3_280322
B.1.1.529 (BA.2.9)	27382	GAT	CTC	MNP	Homozygous	ORF6	QHD43420.1	1	c.181_183delGATinsCTC	p.Asp61Leu	missense_variant	MODERATE	1.0	2945.82	2990	Patient3_280322
B.1.1.529 (BA.2.9)	27807	C	T	SNP	Homozygous	ORF8	QHD43422.1	-1	c.-87C>T	.	upstream_gene_variant	MODIFIER	0.9975	2948.9	7698	Patient3_280322
B.1.1.529 (BA.2.9)	28271	A	T	SNP	Homozygous	N	QHD43423.2	-1	c.-3A>T	.	upstream_gene_variant	MODIFIER	0.9875	2841.76	13796	Patient3_280322
B.1.1.529 (BA.2.9)	28311	C	T	SNP	Homozygous	N	QHD43423.2	1	c.38C>T	p.Pro13Leu	missense_variant	MODERATE	0.995	2352.92	13796	Patient3_280322
B.1.1.529 (BA.2.9)	28361	GGAGAACGCA	G	DEL	Homozygous	N	QHD43423.2	1	c.90_98delAGAACGCAG	p.Glu31_Ser33del	disruptive_inframe_deletion	MODERATE	1.0	2922.71	4049	Patient3_280322
B.1.1.529 (BA.2.9)	28881	GGG	AAC	MNP	Homozygous	N	QHD43423.2	1	c.608_610delGGGinsAAC	p.ArgGly203LysArg	missense_variant	MODERATE	1.0	2959.9	2804	Patient3_280322
B.1.1.529 (BA.2.9)	29510	A	C	SNP	Homozygous	N	QHD43423.2	1	c.1237A>C	p.Ser413Arg	missense_variant	MODERATE	1.0	2944.0	1403	Patient3_280322
B.1.1.529 (BA.2.9)	29733	CGAGGCCACGCGGAGTACGATCGAGTG	C	DEL	Homozygous	S	QHD43416.1	-1	c.*4350_*4375delGAGGCCACGCGGAGTACGATCGAGTG	.	downstream_gene_variant	MODIFIER	1.0	2955.72	749	Patient3_280322
B.1.1.529 (BA.1.15)	241	C	T	SNP	Homozygous	orf1ab	QHD43415.1	-1	c.-25C>T	.	upstream_gene_variant	MODIFIER	0.990566	760.268	106	Patient5_090122
B.1.1.529 (BA.1.15)	2832	A	G	SNP	Homozygous	orf1ab	QHD43415.1	1	c.2567A>G	p.Lys856Arg	missense_variant	MODERATE	0.987437	2824.53	476	Patient5_090122
B.1.1.529 (BA.1.15)	3037	C	T	SNP	Homozygous	orf1ab	QHD43415.1	1	c.2772C>T	p.Phe924Phe	synonymous_variant	LOW	1.0	2939.9	1429	Patient5_090122

B.1.1.529 (BA.1.15)	5386	T	G	SNP	Homozygous	orf1ab	QHD43415.1	1	c.5121T>G	p.Ala1707Ala	synonymous_variant	LOW	1.0	1857.1	249	Patient5_090122
B.1.1.529 (BA.1.15)	6512	AGTT	A	DEL	Homozygous	orf1ab	QHD43415.1	1	c.6248_6250delGTT	p.Ser2083_Leu2084delinsIle	disruptive_inframe_deletion	MODERATE	1.0	2949.42	435	Patient5_090122
B.1.1.529 (BA.1.15)	8393	G	A	SNP	Homozygous	orf1ab	QHD43415.1	1	c.8128G>A	p.Ala2710Thr	missense_variant	MODERATE	1.0	2856.51	385	Patient5_090122
B.1.1.529 (BA.1.15)	10029	C	T	SNP	Homozygous	orf1ab	QHD43415.1	1	c.9764C>T	p.Thr3255Ile	missense_variant	MODERATE	0.9975	2948.66	459	Patient5_090122
B.1.1.529 (BA.1.15)	10135	T	C	SNP	Homozygous	orf1ab	QHD43415.1	1	c.9870T>C	p.Leu3290Leu	synonymous_variant	LOW	1.0	2961.02	495	Patient5_090122
B.1.1.529 (BA.1.15)	10449	C	A	SNP	Homozygous	orf1ab	QHD43415.1	1	c.10184C>A	p.Pro3395His	missense_variant	MODERATE	0.994638	2716.48	373	Patient5_090122
B.1.1.529 (BA.1.15)	11282	AGTTTGTCTG	A	DEL	Homozygous	orf1ab	QHD43415.1	1	c.11022_11030delGTCTGGTTT	p.Leu3674_Gly3676del	disruptive_inframe_deletion	MODERATE	1.0	1088.46	148	Patient5_090122
B.1.1.529 (BA.1.15)	11537	A	G	SNP	Homozygous	orf1ab	QHD43415.1	1	c.11272A>G	p.Ile3758Val	missense_variant	MODERATE	1.0	801.253	108	Patient5_090122
B.1.1.529 (BA.1.15)	13195	T	C	SNP	Homozygous	orf1ab	QHD43415.1	1	c.12930T>C	p.Val4310Val	synonymous_variant	LOW	1.0	2694.18	362	Patient5_090122
B.1.1.529 (BA.1.15)	14408	C	T	SNP	Homozygous	orf1ab	QHD43415.1	2	c.14144C>T	p.Pro4715Leu	missense_variant	MODERATE	1.0	1040.26	140	Patient5_090122
B.1.1.529 (BA.1.15)	15240	C	T	SNP	Homozygous	orf1ab	QHD43415.1	2	c.14976C>T	p.Asn4992Asn	synonymous_variant	LOW	1.0	2982.07	648	Patient5_090122
B.1.1.529 (BA.1.15)	17105	AT	A	DEL	Heterozygous	orf1ab	QHD43415.1	2	c.16845delT	p.Phe5615fs	frameshift_variant	HIGH	0.62766	194.205	284	Patient5_090122
B.1.1.529 (BA.1.15	18163	A	G	SNP	Homozygous	orf1ab	QHD43415.1	2	c.17899A>G	p.Ile5967Val	missense_variant	MODERATE	0.971751	1190.82	177	Patient5_090122

)															
B.1.1.529 (BA.1.15)	21762	CTATACATG	TTA	MIXED	Homozygous	S	QHD43416.1	1	c.200_208delCTATACATGinsTTA	p.Ala67_Val70delinsValIle	missense_variant&disruptive_inframe_deletion	MODERATE	1.0	211.414	28	Patient5_090122
B.1.1.529 (BA.1.15)	22193	AATT	A	DEL	Homozygous	S	QHD43416.1	1	c.632_634delATT	p.Asn211_Leu212delinsIle	disruptive_inframe_deletion	MODERATE	1.0	1531.22	207	Patient5_090122
B.1.1.529 (BA.1.15)	22204	T	TGAGCCAGAA	INS	Homozygous	S	QHD43416.1	1	c.644_645insGCCAGAAGA	p.Arg214_Asp215insGluProGlu	disruptive_inframe_insertion	MODERATE	0.868932	1014.47	207	Patient5_090122
B.1.1.529 (BA.1.15)	22578	G	A	SNP	Homozygous	S	QHD43416.1	1	c.1016G>A	p.Gly339Asp	missense_variant	MODERATE	1.0	253.401	34	Patient5_090122
B.1.1.529 (BA.1.15)	22673	TC	CT	MNP	Homozygous	S	QHD43416.1	1	c.1111_1112delTCinsCT	p.Ser371Leu	missense_variant	MODERATE	1.0	510.853	69	Patient5_090122
B.1.1.529 (BA.1.15)	22679	T	C	SNP	Homozygous	S	QHD43416.1	1	c.1117T>C	p.Ser373Pro	missense_variant	MODERATE	1.0	510.852	69	Patient5_090122
B.1.1.529 (BA.1.15)	22686	C	T	SNP	Homozygous	S	QHD43416.1	1	c.1124C>T	p.Ser375Phe	missense_variant	MODERATE	1.0	385.613	51	Patient5_090122
B.1.1.529 (BA.1.15)	22813	G	T	SNP	Homozygous	S	QHD43416.1	1	c.1251G>T	p.Lys417Asn	missense_variant	MODERATE	1.0	2973.58	477	Patient5_090122
B.1.1.529 (BA.1.15)	22882	T	G	SNP	Homozygous	S	QHD43416.1	1	c.1320T>G	p.Asn440Lys	missense_variant	MODERATE	0.997494	2940.7	984	Patient5_090122
B.1.1.529 (BA.1.15)	22898	G	A	SNP	Homozygous	S	QHD43416.1	1	c.1336G>A	p.Gly446Ser	missense_variant	MODERATE	1.0	2947.71	997	Patient5_090122
B.1.1.529 (BA.1.15)	22992	GCAC	ACAA	MNP	Homozygous	S	QHD43416.1	1	c.1430_1433delGCACinsACAA	p.SerThr477AsnLys	missense_variant	MODERATE	1.0	2942.72	590	Patient5_090122
B.1.1.529 (BA.1.15)	23013	A	C	SNP	Homozygous	S	QHD43416.1	1	c.1451A>C	p.Glu484Ala	missense_variant	MODERATE	0.994987	2347.44	593	Patient5_090122

B.1.1.529 (BA.1.15)	23040	A	G	SNP	Homozygous	S	QHD43416.1	1	c.1478A>G	p.Gln493Arg	missense_variant	MODERATE	1.0	2705.68	576	Patient 5_090122
B.1.1.529 (BA.1.15)	23202	C	A	SNP	Homozygous	S	QHD43416.1	1	c.1640C>A	p.Thr547Lys	missense_variant	MODERATE	1.0	711.711	94	Patient 5_090122
B.1.1.529 (BA.1.15)	23403	A	G	SNP	Homozygous	S	QHD43416.1	1	c.1841A>G	p.Asp614Gly	missense_variant	MODERATE	1.0	2980.45	423	Patient 5_090122
B.1.1.529 (BA.1.15)	23525	C	T	SNP	Homozygous	S	QHD43416.1	1	c.1963C>T	p.His655Tyr	missense_variant	MODERATE	1.0	2981.96	401	Patient 5_090122
B.1.1.529 (BA.1.15)	23599	T	G	SNP	Homozygous	S	QHD43416.1	1	c.2037T>G	p.Asn679Lys	missense_variant	MODERATE	1.0	2065.51	278	Patient 5_090122
B.1.1.529 (BA.1.15)	23604	C	A	SNP	Homozygous	S	QHD43416.1	1	c.2042C>A	p.Pro681His	missense_variant	MODERATE	1.0	2045.23	275	Patient 5_090122
B.1.1.529 (BA.1.15)	23854	C	A	SNP	Homozygous	S	QHD43416.1	1	c.2292C>A	p.Asn764Lys	missense_variant	MODERATE	1.0	2247.86	303	Patient 5_090122
B.1.1.529 (BA.1.15)	23948	G	T	SNP	Homozygous	S	QHD43416.1	1	c.2386G>T	p.Asp796Tyr	missense_variant	MODERATE	1.0	674.512	91	Patient 5_090122
B.1.1.529 (BA.1.15)	24130	C	A	SNP	Homozygous	S	QHD43416.1	1	c.2568C>A	p.Asn856Lys	missense_variant	MODERATE	1.0	1048.06	141	Patient 5_090122
B.1.1.529 (BA.1.15)	24424	A	T	SNP	Homozygous	S	QHD43416.1	1	c.2862A>T	p.Gln954His	missense_variant	MODERATE	0.997494	2373.9	415	Patient 5_090122
B.1.1.529 (BA.1.15)	24469	T	A	SNP	Homozygous	S	QHD43416.1	1	c.2907T>A	p.Asn969Lys	missense_variant	MODERATE	1.0	2978.87	1264	Patient 5_090122
B.1.1.529 (BA.1.15)	24503	C	T	SNP	Homozygous	S	QHD43416.1	1	c.2941C>T	p.Leu981Phe	missense_variant	MODERATE	0.97995	2204.57	1289	Patient 5_090122
B.1.1.529 (BA.1.15	25000	C	T	SNP	Homozygous	S	QHD43416.1	1	c.3438C>T	p.Asp1146Asp	synonymous_variant	LOW	1.0	2982.13	527	Patient 5_090122

)															
B.1.1.529 (BA.1.15)	25584	C	T	SNP	Homozygous	ORF3a	QHD43417.1	1	c.192C>T	p.Thr64Thr	synonymous_variant	LOW	0.984925	2800.48	622	Patient 5_090122
B.1.1.529 (BA.1.15)	25708	C	T	SNP	Homozygous	ORF3a	QHD43417.1	1	c.316C>T	p.Leu106Phe	missense_variant	MODERATE	0.9975	2937.91	474	Patient 5_090122
B.1.1.529 (BA.1.15)	26270	C	T	SNP	Homozygous	E	QHD43418.1	1	c.26C>T	p.Thr9Ile	missense_variant	MODERATE	1.0	2982.16	3575	Patient 5_090122
B.1.1.529 (BA.1.15)	26530	A	G	SNP	Homozygous	M	QHD43419.1	1	c.8A>G	p.Asp3Gly	missense_variant	MODERATE	1.0	474.141	64	Patient 5_090122
B.1.1.529 (BA.1.15)	26709	G	A	SNP	Homozygous	M	QHD43419.1	1	c.187G>A	p.Ala63Thr	missense_variant	MODERATE	1.0	2974.78	454	Patient 5_090122
B.1.1.529 (BA.1.15)	27259	A	C	SNP	Homozygous	ORF6	QHD43420.1	1	c.58A>C	p.Arg20Arg	synonymous_variant	LOW	0.996845	2330.22	317	Patient 5_090122
B.1.1.529 (BA.1.15)	27807	C	T	SNP	Homozygous	ORF8	QHD43422.1	-1	c.-87C>T	.	upstream_gene_variant	MODIFIER	1.0	920.258	124	Patient 5_090122
B.1.1.529 (BA.1.15)	28271	A	T	SNP	Homozygous	N	QHD43423.2	-1	c.-3A>T	.	upstream_gene_variant	MODIFIER	0.9875	2768.04	2299	Patient 5_090122
B.1.1.529 (BA.1.15)	28311	C	T	SNP	Homozygous	N	QHD43423.2	1	c.38C>T	p.Pro13Leu	missense_variant	MODERATE	0.99	2301.98	2308	Patient 5_090122
B.1.1.529 (BA.1.15)	28361	GGAGAACGCA	G	DEL	Homozygous	N	QHD43423.2	1	c.90_98delAGAACGCAG	p.Glu31_Ser33del	disruptive_inframe_deletion	MODERATE	1.0	2921.2	766	Patient 5_090122
B.1.1.529 (BA.1.15)	28881	GGG	AAC	MNP	Homozygous	N	QHD43423.2	1	c.608_610delGGGinsAAC	p.ArgGly203LysArg	missense_variant	MODERATE	0.992424	2861.8	641	Patient 5_090122
B.1.1.529 (BA.1.15)	29301	A	G	SNP	Homozygous	N	QHD43423.2	1	c.1028A>G	p.Asp343Gly	missense_variant	MODERATE	0.994382	1298.47	178	Patient 5_090122

B.1.1.529 (BA.1.15)	29421	C	T	SNP	Homozygous	N	QHD43423.2	1	c.1148C>T	p.Pro383Leu	missense_variant	MODERATE	0.99569	1698.07	232	Patient 5_090122

Supplementary Table 2: complete list of mutations identified in the SARS-CoV-2 genomes from patients described.
